# Selection and Characterization of Vaginal Lactobacilli from Healthy Algerian Women with Potential Probiotic Properties Against Urogenital Pathogens

**DOI:** 10.3390/antibiotics15090875

**Published:** 2026-09-07

**Authors:** Asma Mammar, Hayat Trabsa, Stefania Dentice Maidana, Ammar Ayachi, Asma Abdessemed, Leonardo Albarracin, Mariano Elean, Kamel Boubakri, Ayelen A. Baillo, Haruki Kitazawa, Julio Villena

**Affiliations:** 1 Laboratory of Promotion of Agricultural Innovation in Arid Regions, University of Biskra, Biskra 07000, Algeria; asma.mammar@univ-biskra.dz; 2 Department of Natural and Life Sciences, Faculty of Natural and Life Sciences, Earth and Universe Sciences, University of Biskra, Biskra 07000, Algeria; hayat.trabsa@univ-biskra.dz; 3 Laboratory of Applied Biochemistry, Faculty of Nature and Life Sciences, University of Setif 1, Setif 19000, Algeria; 4 Food and Feed Immunology Group, Laboratory of Animal Food Function, Graduate School of Agricultural Science, Tohoku University, Sendai 980-8572, Japan; stefania.dentice.maidana.b8@tohoku.ac.jp; 5 Laboratory of Respiratory Immunology (LaRI), Division of Animal Immunology and Omics, International Education and Research Centre for Food and Agricultural Immunology (CFAI), Graduate School of Agricultural Science, Tohoku University, Sendai 980-8572, Japan; albarracin.leonardo.miguel.e5@tohoku.ac.jp; 6 Department of Veterinary Sciences, Institute of Veterinary Sciences and Agronomic Sciences, University of Batna 1, Batna 05000, Algeria; ammar.ayachi@univ-batna.dz; 7 Mother and Child Specialized Hospital (EHS), Batna 05000, Algeria; asma.abdessemed@univ-batna2.dz; 8 Laboratory of Immunobiotechnology, Reference Centre for Lactobacilli (CERELA-CONICET), San Miguel de Tucumán CP4000, Argentina; melean@cerela.org.ar (M.E.); abaillo@cerela.org.ar (A.A.B.); 9 Department of Biology and Ecology, Faculty of Sciences, University of Médéa, Médéa 26000, Algeria; boubakri.kamel@univ-medea.dz; 10 Livestock Immunology Unit, International Education and Research Centre for Food and Agricultural Immunology (CFAI), Graduate School of Agricultural Science, Tohoku University, Sendai 980-8572, Japan

**Keywords:** vaginal microbiota, lactic acid bacteria, probiotic, Algerian women

## Abstract

**Background:** The vaginal microbiota of healthy women is predominantly composed of lactic acid bacteria (LAB), particularly species from the lactobacilli group, which contribute to the maintenance of vaginal health through multiple protective mechanisms. **Objectives:** In the present study, vaginal LAB isolated from healthy premenopausal Algerian women were evaluated for their probiotic potential. **Methods**: A total of 259 LAB isolates were initially screened for antimicrobial activity against vaginal pathogens, among which 164 strains exhibited inhibitory effects. Based on antimicrobial capacity and biofilm formation ability, 38 strains were selected for further characterization. A refined subset of 15 strains belonging to *Lactiplantibacillus (Lpb.) plantarum*, *Limosilactobacillus (Lmb.) fermentum*, and *Ligilactobacillus (Lgb.) salivarius* were subsequently identified according to their superior antimicrobial and probiotic-related properties. The selected strains were evaluated for several functional and safety characteristics, including antimicrobial activity, production of lactic acid and hydrogen peroxide (H_2_O_2_), biofilm formation, auto-aggregation, cell surface hydrophobicity, antibiotic susceptibility, hemolytic activity, and tolerance to acidic conditions. These studies were complemented with comparative genomics using the complete genome sequences of selected strains. **Results**: The isolates demonstrated significant inhibitory activity against vaginal pathogens associated with aerobic vaginitis, and vulvovaginal candidiasis. In addition, strong correlations were observed between metabolite production and antimicrobial activity. Several strains also exhibited enhanced biofilm formation, aggregation capacity, and favorable safety profiles. Among the tested isolates, *Lmb. fermentum* 70BJ2, *Lpb. plantarum* 87JG8, and *Lpb.plantarum* N17 emerged as particularly promising probiotic candidates due to their combined antimicrobial, metabolic, and adhesion-related properties. **Conclusions**: Overall, these findings demonstrate that the vaginal microbiota of healthy Algerian women represents a valuable source of potential probiotic strains and provide a strong basis for future in vivo validation and clinical application in the prevention and management of vaginal infections.

## 1. Introduction

A balanced vaginal microbiota plays a pivotal role in maintaining a healthy cervicovaginal environment and is essential for optimal functioning of the female reproductive system [1,2,3]. However, despite the global importance that has been given to studying host–microbe interactions in the human vaginal tract, current microbiome datasets remain heavily skewed toward Western populations, with limited representation from other regions of the world [4]. The importance of evaluating microbiota from different geographic and cultural contexts lies in capturing the true global diversity of microbial life associated with human beings [5]. Existing data are insufficient to draw universal conclusions about microbiome–health relationships.

Research from sub-Saharan Africa similarly reports a high prevalence of heterogeneous consortia of non-lactobacilli communities [6]. In contrast, North Africa remains largely understudied, with only a few reports available from Morocco, Tunisia, and Algeria describing variable lactobacilli dominance and mixed microbial profiles [7,8,9]. This lack of available data reflects a broader limitation in microbiome research, as current datasets are predominantly focused on Western populations, while other regions remain underrepresented. Consequently, investigating the vaginal microbiota of Algerian women is important to address this regional knowledge gap and to contribute to a more comprehensive and globally representative understanding of vaginal microbial diversity.

Unlike other body sites where high microbial diversity is indicative of health, a healthy vaginal niche requires a balanced microbiota with low diversity, dominated by lactobacilli (the former genus *Lactobacillus*). However, various factors such as age, hormonal levels, sexual practices, lifestyle, and hygiene habits can disrupt this balance, leading to ’’dysbiosis’’ which is characterized by a reduced abundance of *Lactobacillus* accompanied by increased microbial diversity. This state may further progress to “pathobiosis”, wherein opportunistic pathogens increase. Both dysbiosis and pathobiosis states are strongly correlated with the development of several vaginal diseases, including bacterial vaginosis, aerobic vaginitis, and vulvovaginal candidiasis, as well as with a higher susceptibility to sexually transmitted infections (STIs) [10,11,12]. The conventional treatment of genital infections primarily relies on the use of antibiotics and antifungal agents. However, the long-term and repeated use of these therapies has led to treatment failures, particularly when persistent pathogen biofilm formation and recurrent microbial communities are present [12,13,14]. These treatments have also increased the prevalence of antibiotic resistance and recurrent infections, which further complicates the effective management of these pathologies [15].

Species belonging to the lactobacilli group are widely recognized as key biomarkers dominating healthy vaginal ecosystems. Particularly during reproductive age, the most common species inhabiting the female genital tract include *Lactobacillus (Lb.) crispatus*, *Lb. iners*, *Lb. gasseri*, and *Lb. jensenii* [16,17,18,19]. However, other lactobacilli, including *Lpb. plantarum*, *Lmb. fermentum*, and *Lgb. salivarius*, have also been isolated from the human vaginal tract and described as potential probiotic candidates due to their antimicrobial and colonization properties [20,21]. These species possess several protective functions against vaginal infections. The production of antimicrobial compounds, particularly lactic acid, which is generated by converting glycogen-derived substrates into L- and D-lactic acid under anaerobic conditions, has been associated with the control of pathogens in the vaginal mucosa. Lactic acid helps maintain a low vaginal pH < 4.5, creating an acidic microenvironment that serves as a physiological defense mechanism against pathogenic colonization [3,22]. Additionally, lactobacilli species can synthesize hydrogen peroxide and bacteriocins that contribute to their microbicidal activities [23]. They can also produce biosurfactant compounds that act as anti-biofilm agents [24]. Another major mechanism involved in the protective capacities of lactobacilli is their ability to inhibit the adhesion of pathogens to the vaginal epithelium. This competitive exclusion allows lactobacilli to reduce the binding and colonization of vaginal epithelial cells by pathogens, which not only reduces the space for growth but also masks epithelial receptors through biofilm formation [25,26].

For these reasons, the use of lactobacilli strains for the preservation or the restoration of beneficial balanced vaginal microbiota has been emphasized by scientists [27,28,29]. For example, ref. [20] identified vaginal strains of *Lpb. plantarum*, *Lgb. salivarius*, and *Lmb. fermentum* isolated from healthy women that exhibited strong antimicrobial activity, biofilm formation, and adhesion-related properties, highlighting their potential as probiotic candidates for vaginal health. In addition, [28] reported that specific probiotic lactobacilli, including *Lacticaseibacillus (Lcb.) rhamnosus* GR-1 and *Lmb. reuteri* RC-14, were associated with improved vaginal microbiota composition and reduced recurrence of vaginal infections. According to [30], only a few well-characterized lactobacilli strains have strong evidence for urogenital probiotic use. One example is *Lcb. rhamnosus* GR-1, a clinically validated strain that can be administered orally or vaginally and supports restoration of a lactobacilli-dominated microbiota. Another important example is *L. crispatus* CTV-05, which is used vaginally to promote lactic-acid–rich conditions and reduce recurrence of bacterial vaginosis. Importantly, beneficial probiotic effects are considered strain-specific; therefore, the identification and functional characterization of novel vaginal lactobacilli isolates remains essential for the development of effective probiotic strategies aimed at preventing and controlling urogenital infections in specific populations, such as Algerian women.

In this study, LAB from the vaginal tract of premenopausal Algerian women residing in Batna city were isolated and characterized regarding their probiotic potential. The isolated vaginal LAB strains were evaluated for their capacity to inhibit the growth of vaginal pathogens, and the potential factors involved in their antimicrobial activities were evaluated, including their ability to produce hydrogen peroxide and lactic acid, cell surface hydrophobicity, biofilm formation, and auto-aggregation capacity. These studies were complemented with a comparative genomic evaluation using the complete genome sequences of the selected strains.

## 2. Results

### 2.1. Isolation and Identification of Vaginal LAB Strains

A total of 259 isolates obtained from the vaginal samples of 160 Algerian women were identified as LAB based on phenotypic characterization. These included 95 long bacilli, 109 short bacilli, and 55 coco-bacilli. The antimicrobial activity test and the biofilm formation test allowed the selection of 15 strains, which were subsequently identified by 16S rRNA gene sequencing analysis (Table 1).

### 2.2. Antimicrobial Activity of Vaginal LAB Strains

**Figure 1:**
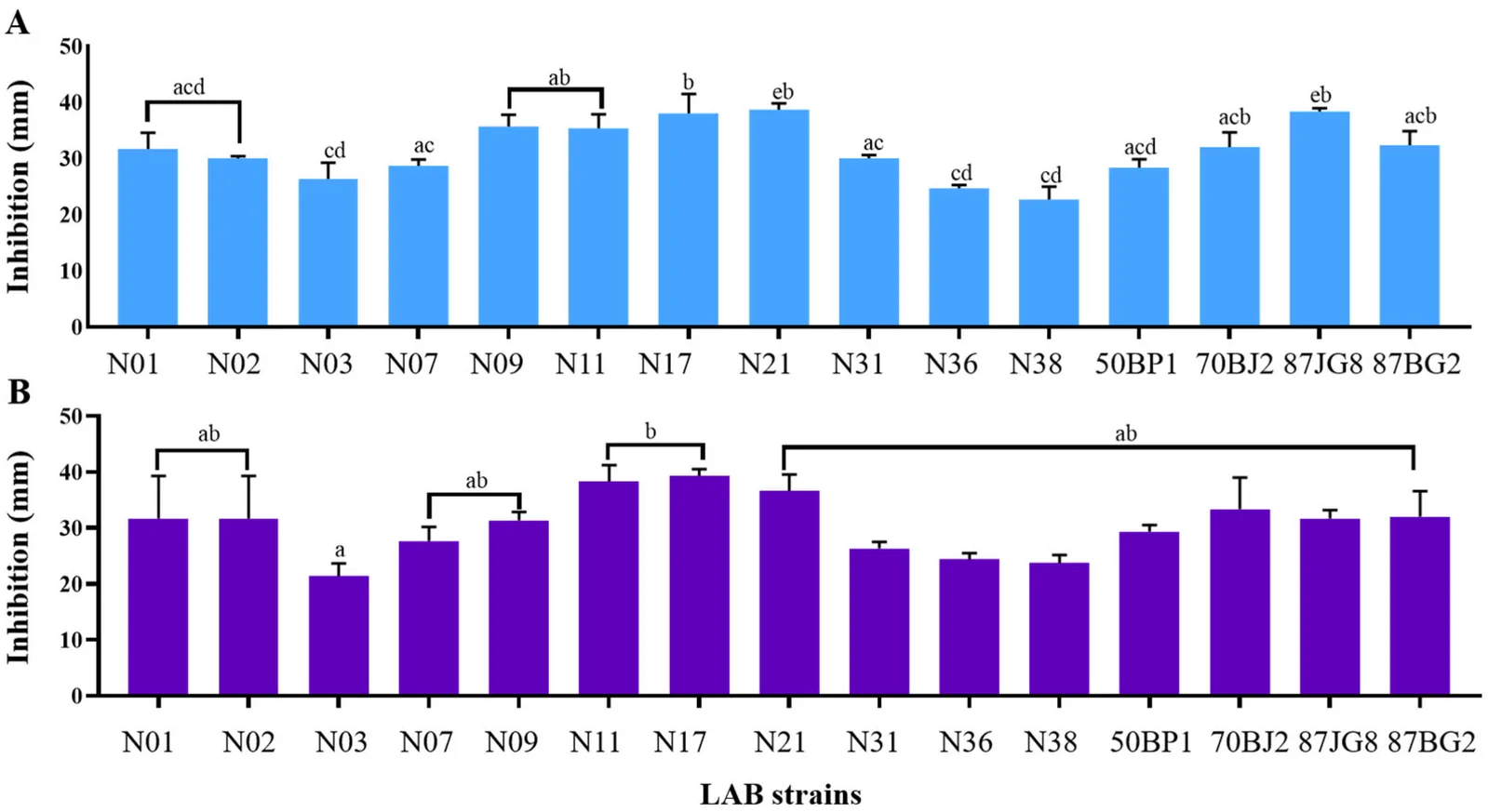
Growth inhibition (mm) by live vaginal LAB strains against (**A**) *E. coli* and (**B**) *K. pneumoniae*. *Lpb. plantarum* (N01, N02, N03, N07, N11, N17, N21, N36, 50BP1, 87JG8, 87BG2); *Lmb. fermentum* (N09, N31, 70BJ2); *Lgb. salivarius* (N03, N38). Bars represent mean values, and error bars indicate the standard deviations (SD) of triplicate inhibition zone measurements. One-way ANOVA (*p* < 0.05 and 95% CI) was applied for single pathogen, followed by Tukey’s post hoc test for comparison across pathogen pairs. Bars that do not share the same letters differ significantly (*p* < 0.05).

**Figure 2:**
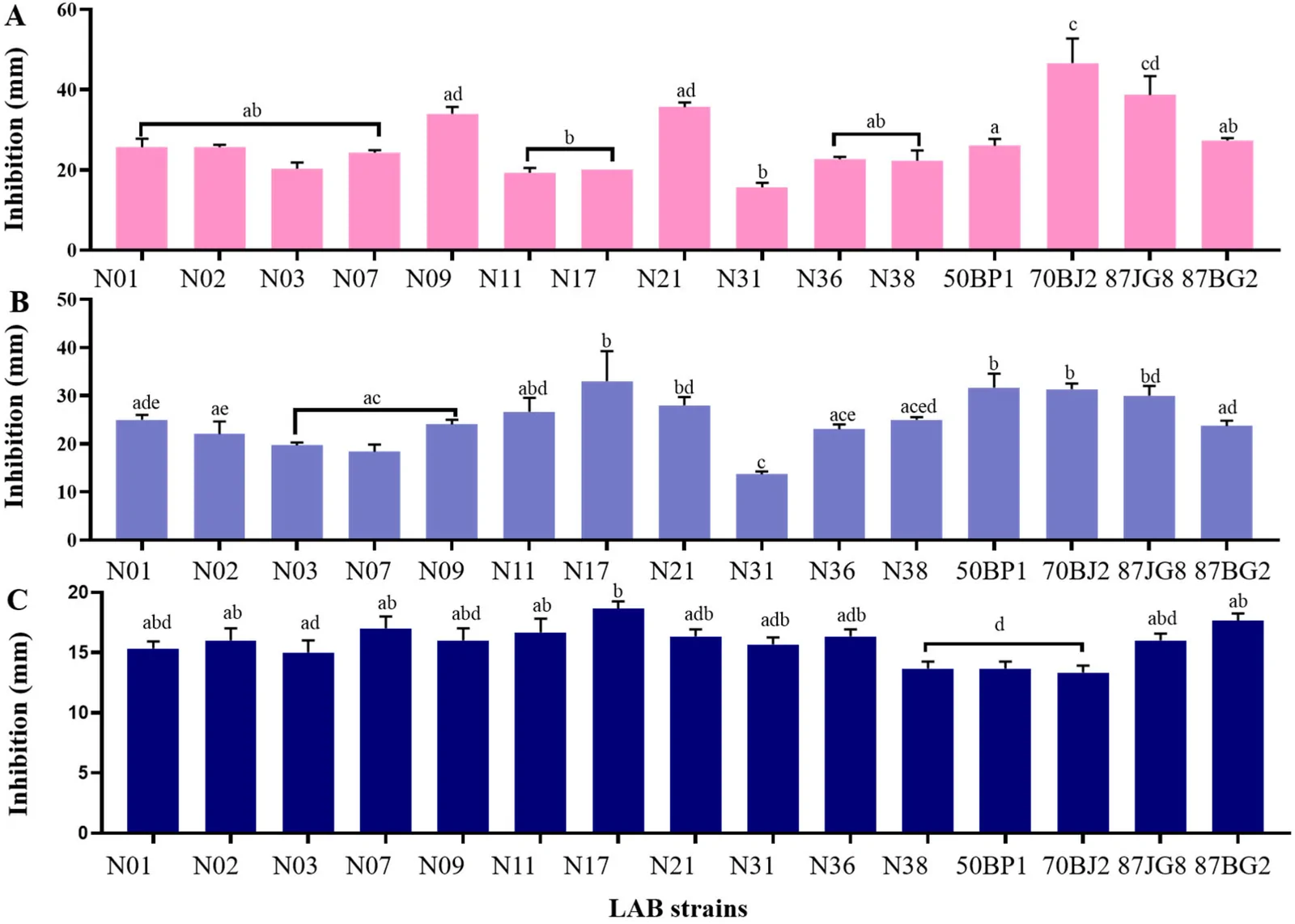
Growth inhibition (mm) by live vaginal LAB strains against (**A**) *S. aureus*, (**B**) *St. agalactiae*, (**C**) *C. albicans*. *Lpb. plantarum* (N01, N02, N03, N07, N11, N17, N21, N36, 50BP1, 87JG8, 87BG2); *Lmb. fermentum* (N09, N31, 70BJ2); *Lgb. salivarius* (N03, N38). Bars represent mean values, and error bars indicate the standard deviations (SD) of triplicate inhibition zone measurements. One-way ANOVA (*p* < 0.05 and 95% CI) was applied for single pathogens, followed by Tukey’s post hoc test for comparison across pathogen pairs. Bars that do not share the same letters differ significantly (*p* < 0.05).

Isolated LAB strains were evaluated for their inhibitory activity against distinct urogenital pathogenic microorganisms, including *Escherichia (E.) coli*, *Klebsiella (K.) pneumoniae*, *Staphylococcus (S.) aureus*, *Streptococcus (St.) agalactiae*, and *Candida (C.) albicans*. Only 164 strains exhibited measurable inhibition zones for bacterial pathogens, while 38 vaginal LAB strains had antifungal activity. The inhibition capacity of these 38 LAB strains is shown in Supplementary Figures S1–S3. Among them, 15 LAB strains showed a remarkable capacity to inhibit the urogenital pathogens *E. coli*, *K. pneumoniae* (Figure 1), *S. aureus*, *St. agalactiae*, and *C. albicans* (Figure 2). *E. coli* was the most susceptible pathogen, with an average inhibition zone of 30.5 mm, followed closely by *K. pneumoniae* (average 29.6 mm) and *S. aureus* (average 24.71 mm). On the other hand, *St. agalactiae* showed moderate sensitivity (average 22.8 mm) to LAB strains, whereas *C. albicans* exhibited the lowest susceptibility, with an average inhibition zone of 14.35 mm. Moreover, the average inhibition zone for Gram-negative pathogens was approximately 6.72 mm larger than for Gram-positive pathogens, indicating that vaginal LAB strains were generally more effective against Gram-negative microorganisms.

Notably, among all these tested LAB, the strains 70BJ2, N17, and 87BG2 demonstrated the strongest antimicrobial capacity.

### 2.3. Production of Lactic Acid and H_2_O_2_ by Vaginal LAB Strains

**Figure 3:**
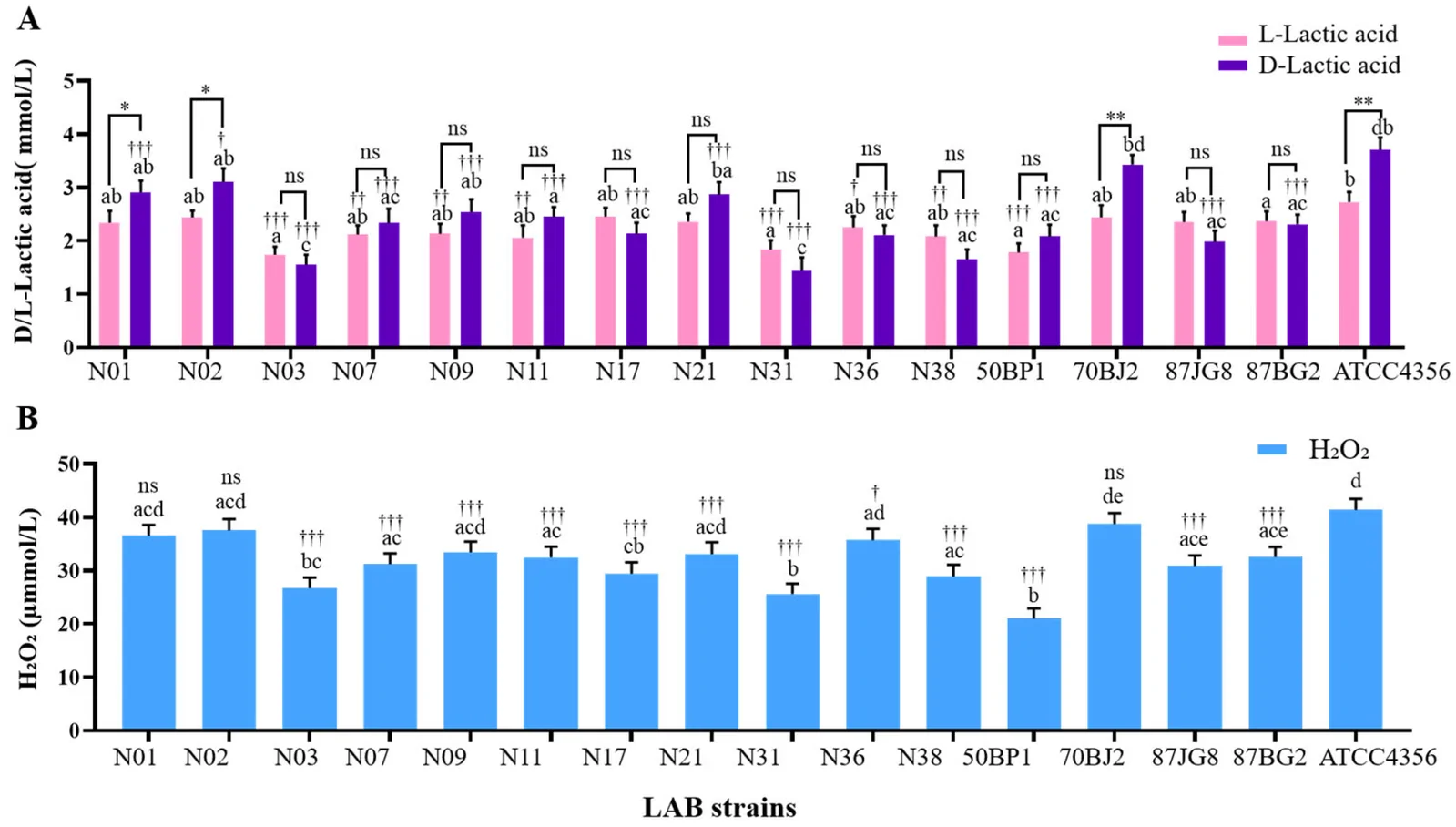
L-lactate, D-lactate (**A**), and hydrogen peroxide production (**B**) by individual vaginal LAB strains. *Lpb. plantarum* (N01, N02, N03, N07, N11, N17, N21, N36, 50BP1, 87JG8, 87BG2); *Lmb. fermentum* (N09, N31, 70BJ2); *Lgb. salivarius* (N03, N38), and *Lb.acidophilus* ATCC 4356 was included as the reference control strain. Bars represent mean values, and error bars indicate the standard deviations (SD) of triplicate measurements. For comparisons among strains, One-way ANOVA followed by Tukey’s post hoc test was applied for pairwise comparisons across strains; bars of the same color that do not share common letters are significantly different (*p* < 0.05; 95% CI). Dunnett’s post hoc test was used to compare individual strains with the reference control (*Lb. acidophilus* ATCC 4356); symbols denote a statistically significant difference († *p* < 0.05, †† *p* < 0.01, ††† *p* < 0.001). For panel A, L- and D-lactic acid production within each strain was compared using a two-tailed *t*-test, which indicates a significant difference * *p* < 0.05, ** *p* < 0.01, ns = not significant.

The production of L-and D- lactic acid was measured for the 38 selected vaginal LAB strains. Supplementary Figure S4A shows the results for all the strains, while Figure 3A shows the results for the 15 strains with the highest antimicrobial activities. L-lactic acid levels ranged from 1.54 to 2.45 mmol/L. In addition, D-lactic acid levels ranged from 1.45 to 3.43 mmol/L. The highest amounts of L-lactate and D-lactate were produced by *Lpb. plantarum* N17 and *Lmb. fermentum* 70BJ2, respectively. In both cases, no significant differences were found when compared to *L. acidophilus* ATCC 4356 (*p* > 0.886; *p* > 0.959).

The production of H_2_O_2_ was also measured for all the selected LAB strains (Figure 3B and Supplementary Figure S4B). H_2_O_2_ concentrations ranged from 20.31 to 38.71 µmol/L. The highest amount was observed for *Lmb. fermentum* 70BJ2, with no-significant differences (*p* > 0.960) compared to the positive control strain (41.35 µmol/L).

**Figure 4:**
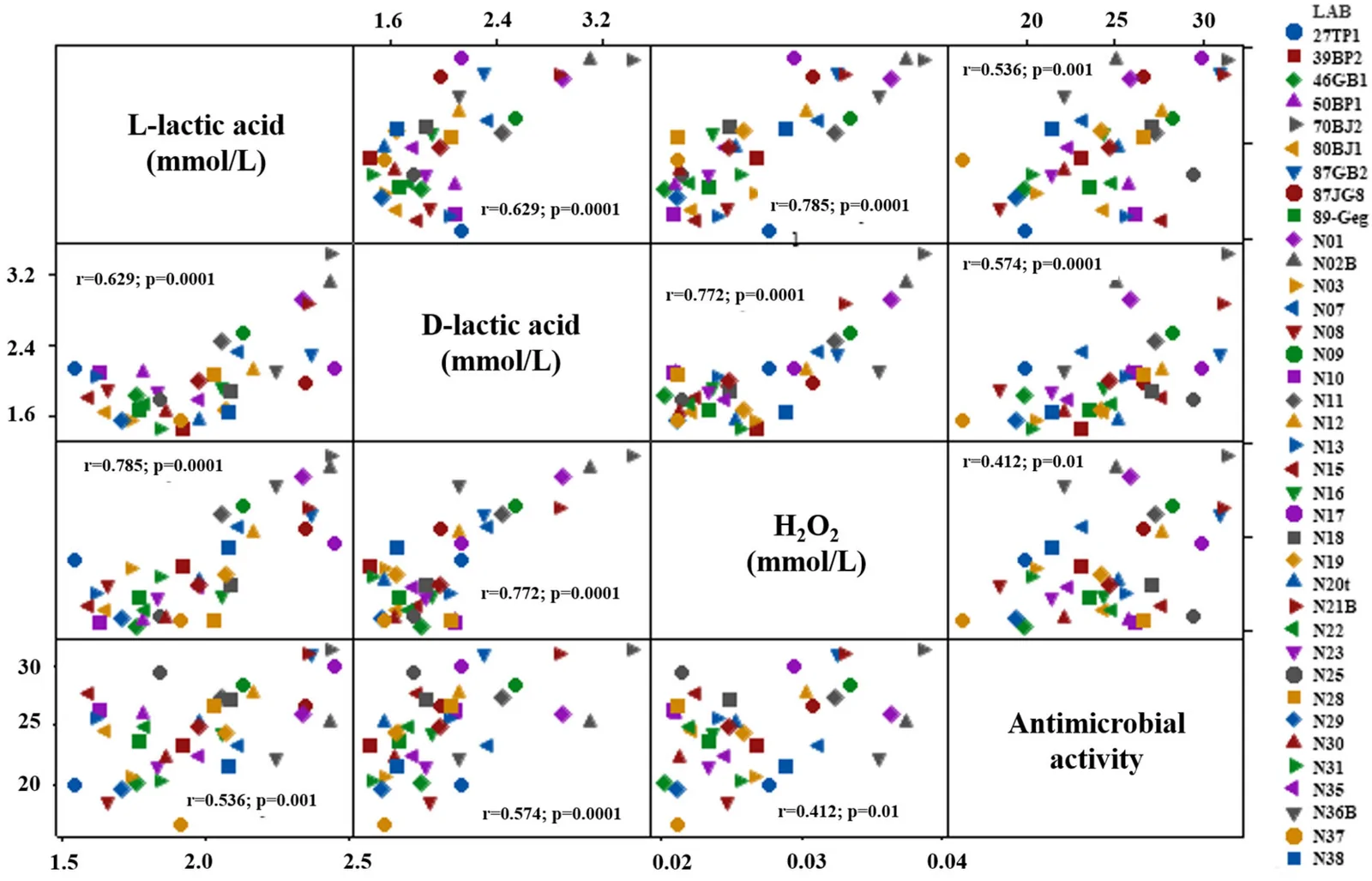
Matrix plot of the correlation (Pearson’s *r* and *p* values) between the three vaginal LAB metabolites (L-lactate, D-lactate, and hydrogen peroxide; see Figure 1) and their mean inhibitory activity against the five pathogens. Individual strains are shown in different colors and shapes.

A two-way ANOVA revealed highly significant differences among the lactic acid metabolites, and the H_2_O_2_ metabolite was evaluated (*p* < 0.001), whereas no significant differences were observed among the strains (*p* > 0.999) (Figure 3). Tukey’s multiple-comparison test showed that H_2_O_2_ production was significantly higher than both L- and D-lactic acid levels (*p* < 0.001), while no significant differences were detected between the two lactic acid isomers (*p* = 0.974). Correlation analysis demonstrated strong positive associations between H_2_O_2_ and L-lactic acid (*r* = 0.785, *p* = 0.0001), as well as between H_2_O_2_ and D-lactic acid (*r* = 0.772, *p* = 0.0001). A significant positive correlation was also observed between L- and D-lactic acid production (*r* = 0.629, *p* = 0.0001), indicating that strains producing high levels of one isomer also tended to produce elevated levels of the other (Figure 4). Overall, these findings suggest that the selected LAB strains produce both lactic acid isomers at comparable levels without a clear preference for either form. Notably, strains 70BJ2, N02, N01, and N21B exhibited the strongest metabolite-associated correlations, particularly between L-lactic acid and H_2_O_2_ production.

### 2.4. Biofilm Formation of Vaginal LAB Strains

**Figure 5:**
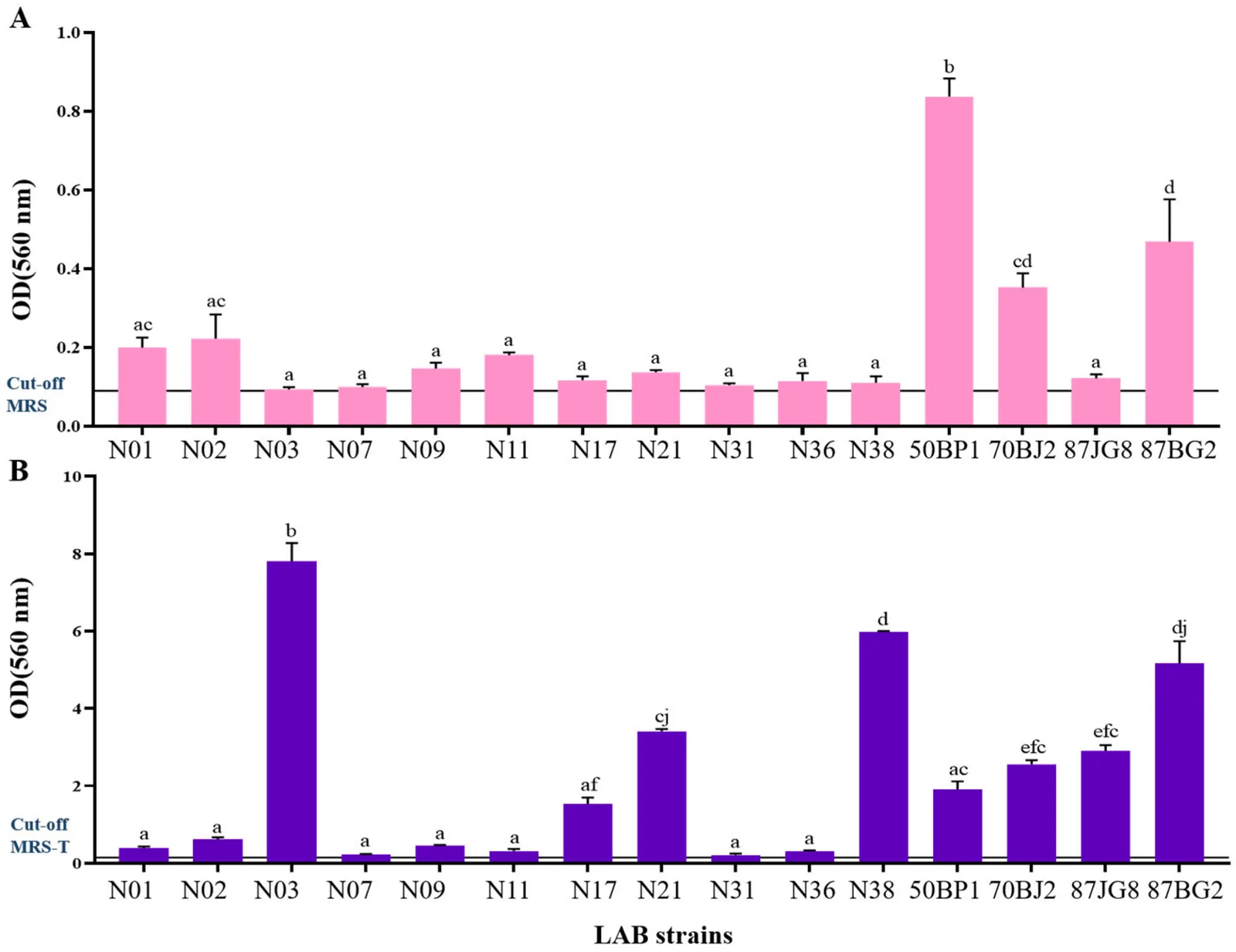
Biofilm formation by vaginal LAB strains in MRS (**A**) and MRS-T (**B**). *Lpb. plantarum* (N01, N02, N03, N07, N11, N17, N21, N36, 50BP1, 87JG8, 87BG2); *Lmb. fermentum* (N09, N31, 70BJ2); *Lgb. salivarius* (N03, N38). Bars represent mean values, and error bars indicate the SD from triplicate experiments; lines indicate the respective cut-off values. Two-way ANOVA with *p* < 0.05 and a 95% confidence interval was used, followed by Tukey‘s multiple comparison test between the groups. Bars of the same color that do not share common letters are significantly different (*p* < 0.05).

The biofilm formation ability of vaginal LAB strains was assessed in both MRS and MRS without Tween 80 (MRS-T) media (Figure 5 and Supplementary Figure S4).

The analysis revealed that the influence of medium composition on biofilm formation varied across strains. In MRS medium, most strains were classified as weak (55.2%) and moderate (34.2%) biofilm producers. Only a small number of LAB strains exhibited strong (7.9%) and very strong (2.6%) biofilm production capacities (Figure 5A and Supplementary Figure S4A). Of note, none of the strains were identified as non-adherent. In contrast, in MRS-T medium, the proportion of strong biofilm producers increased markedly to 13.1% and very strong producers to 31.5%, while weak and moderate producers decreased to 18.4% and 36.8%, respectively (Figure 5B and Supplementary Figure S4B). These results suggest that the removal of Tween 80 promotes stronger biofilm formation in a substantial proportion of the tested strains.

### 2.5. Auto-Aggregation and Hydrophobicity Abilities of Vaginal LAB Strains

**Figure 6:**
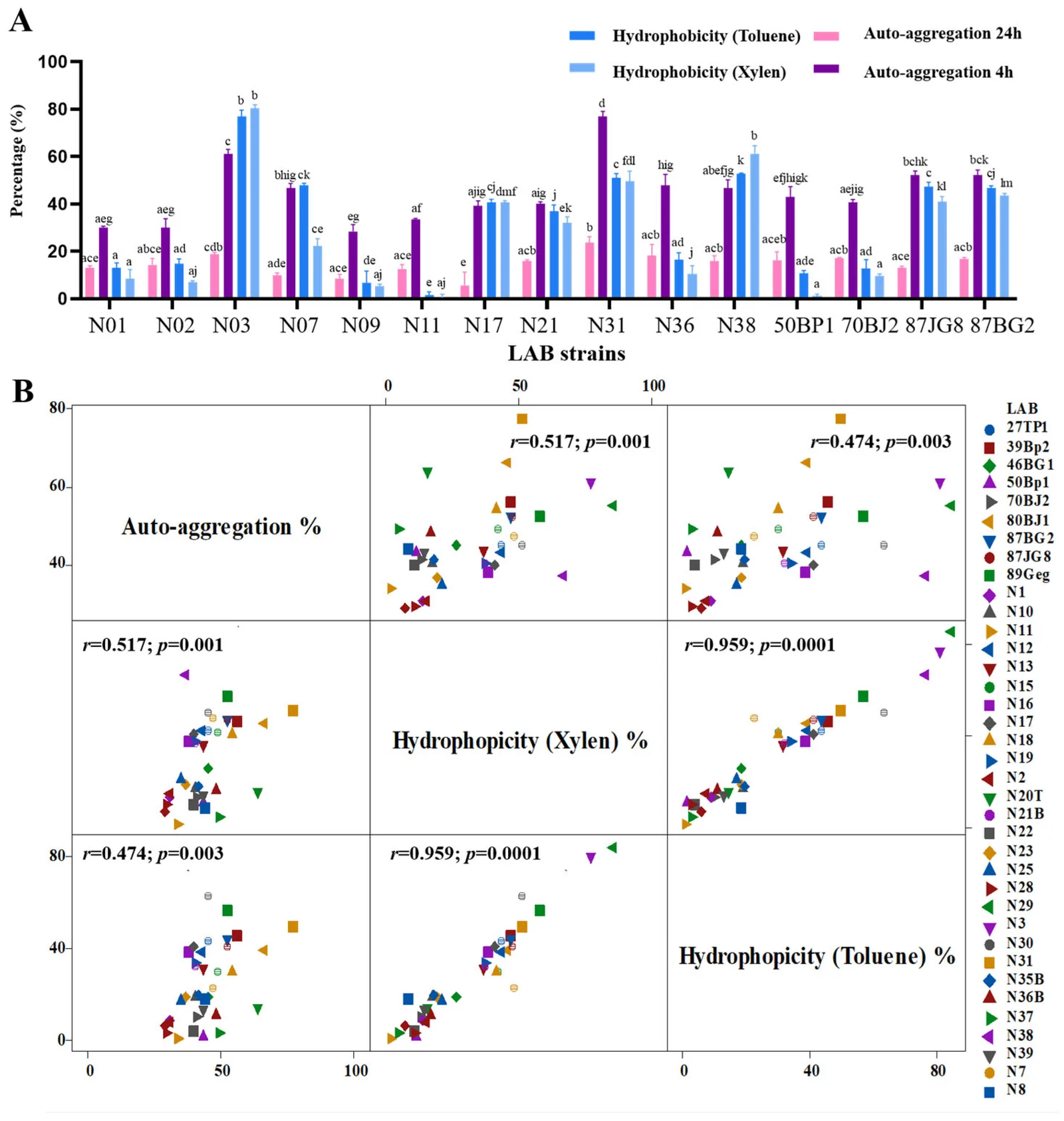
(**A**) Cell surface hydrophobicity to xylene and toluene, and auto-aggregation abilities of vaginal LAB strains at 4 h and 24 h. *Lpb. plantarum* (N01, N02, N03, N07, N11, N17, N21, N36, 50BP1, 87JG8, 87BG2); *Lmb. fermentum* (N09, N31, 70BJ2); *Lgb. salivarius* (N03, N38). Bars represent the mean ± SD from three repetitions. (**B**) Matrix plot showing the correlation (Pearson’s r and *p* values) between hydrophobicity and auto-aggregation in 24 h characterization. One-way ANOVA (*p* < 0.05 and 95% CI) was used to evaluate auto-aggregation of LAB strains at 4 h and 24 h and hydrophobicity toward each solvent. Followed by Tukey’s post hoc test for multiple pairwise comparisons among factors. Bars not sharing the same letters differ significantly (*p* < 0.05). Individual strains are shown in different colors and shapes.

Since adhesion to the vaginal epithelium is considered an important characteristic for probiotic colonization and persistence in the urogenital tract, the auto-aggregation ability and cell surface hydrophobicity of the selected LAB strains were evaluated (Figure 6).

The results of this study revealed significant effects of incubation time and strains on auto-aggregation. Most of the vaginal LAB strains presented approximately 2.5-fold higher auto-aggregation after 24 h compared with 4 h (Supplementary Figure S5). Of note, the strains N31, N20, and N3 showed the highest percentages of auto-aggregation (Figure 6A). When the cell surface hydrophobicity was evaluated, it was observed that most of the strains showed no significant differences when solvents were compared (Supplementary Figure S6). Notably, the strains N38, N31, N29, and N3 displayed the highest hydrophobicity toward both toluene and xylene (Figure 6A).

Pearson correlation analysis revealed significant positive associations between auto-aggregation capacity and cell surface hydrophobicity measured with both xylene (*r* = 0.517, *p* = 0.001) and toluene (*r* = 0.474, *p* = 0.003) (Figure 6B). In addition, a very strong positive correlation was observed between hydrophobicity values obtained with xylene and toluene (*r* = 0.959, *p* = 0.0001), indicating high consistency between both assays for the evaluation of bacterial surface properties. Overall, these results suggest that strains with higher hydrophobicity tend to exhibit greater auto-aggregation capacity, supporting a potential relationship between cell surface characteristics and bacterial adhesion-associated traits.

### 2.6. Tolerance to Low pH

The tolerance to acidic conditions of the selected vaginal LAB strains was evaluated (Table 2 and Supplementary Table S1).

All the LAB strains demonstrated to possess a strong ability to tolerate pH 4.5, since the viability for all of them was superior to 68% after 24 h of incubation. However, after 24 h of incubation at pH 3.5, the survival rates ranged from 3.7% to 45.3%, with the highest value observed in strain N12. At the more extreme pH (2.5), which was evaluated only for 3 h to simulate gastric transit time, significant strain-dependent variation in viability was observed. The highest survival rate was recorded for *Lpb. plantarum* 87GJ8 (92.7%), followed by *Lmb. fermentum* 70BJ2 (91.8%) and N11 (85.6%).

### 2.7. Security Profile of Vaginal LAB Strains

**Figure 7:**
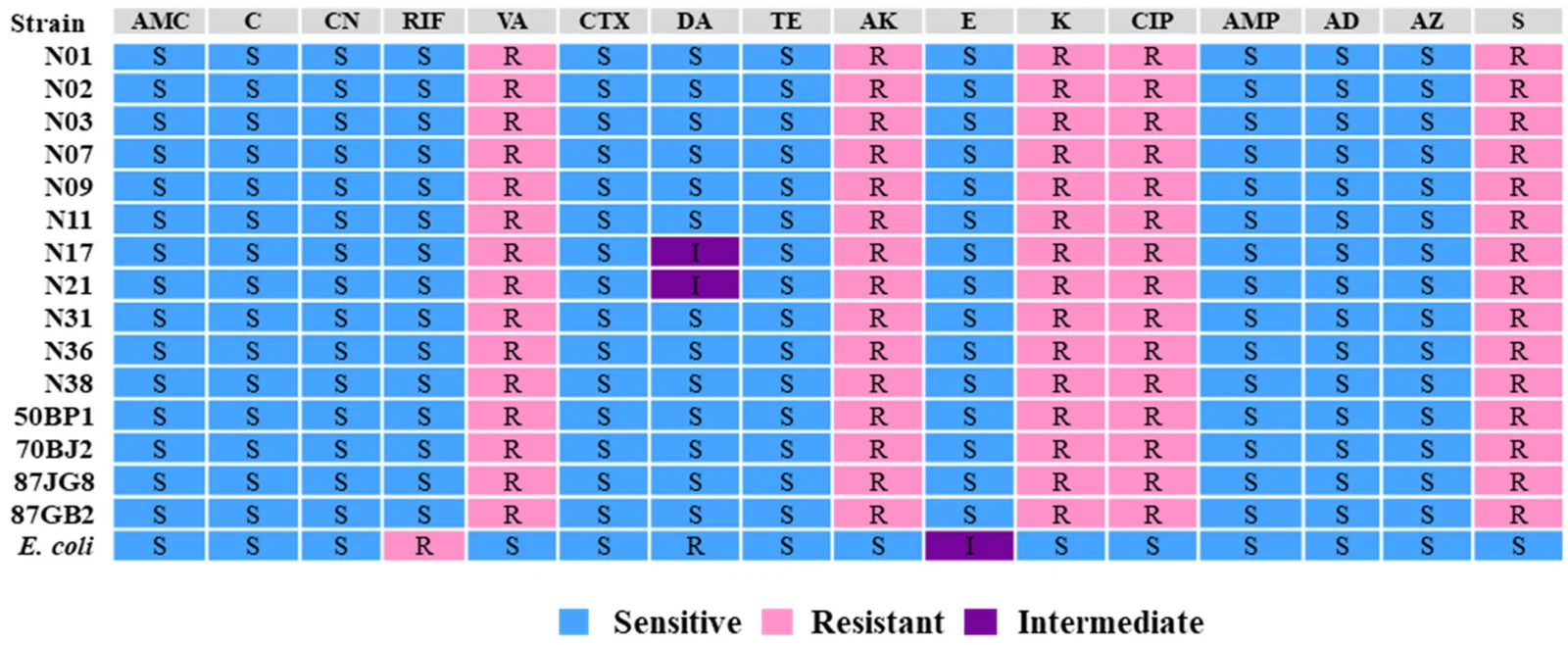
Antibiotic susceptibility and hemolytic activity of vaginal LAB strains on MRS and sheep blood agar. Ranges of zone inhibition diameters exhibited by vaginal LAB strains considered Sensitive (S), Intermediate (I), Resistant (R) based on the interpretive zone diameters outlined in the antimicrobial disk susceptibility test standards [31,32].

The antibiotic susceptibility profile and hemolytic activity of the selected vaginal LAB strains were evaluated as part of their safety assessment for potential probiotic application (Figure 7). All LAB isolates (100%) were susceptible to amoxicillin/clavulanic acid, chloramphenicol, gentamicin, rifampicin, tetracycline, ampicillin, azithromycin, and doxycycline. In contrast, all strains exhibited resistance to vancomycin, kanamycin, ciprofloxacin, and streptomycin. Regarding cefotaxime, most strains (92.1%) were susceptible, whereas 7.8% showed intermediate susceptibility. Similarly, 84.2% of strains were susceptible to clindamycin, 13.1% displayed intermediate susceptibility, and only 2.63% were resistant. For erythromycin, 97.3% of the isolates were susceptible, while 2.6% exhibited intermediate susceptibility. Hemolytic activity assays showed that all LAB strains were non-hemolytic (δ-hemolysis), supporting their safety as potential probiotic candidates (Figure 7).

### 2.8. Phenotypic Relationships Among the Vaginal LAB Strains Selected LAB Strains Based on Probiotic-Related Variables

**Figure 8:**
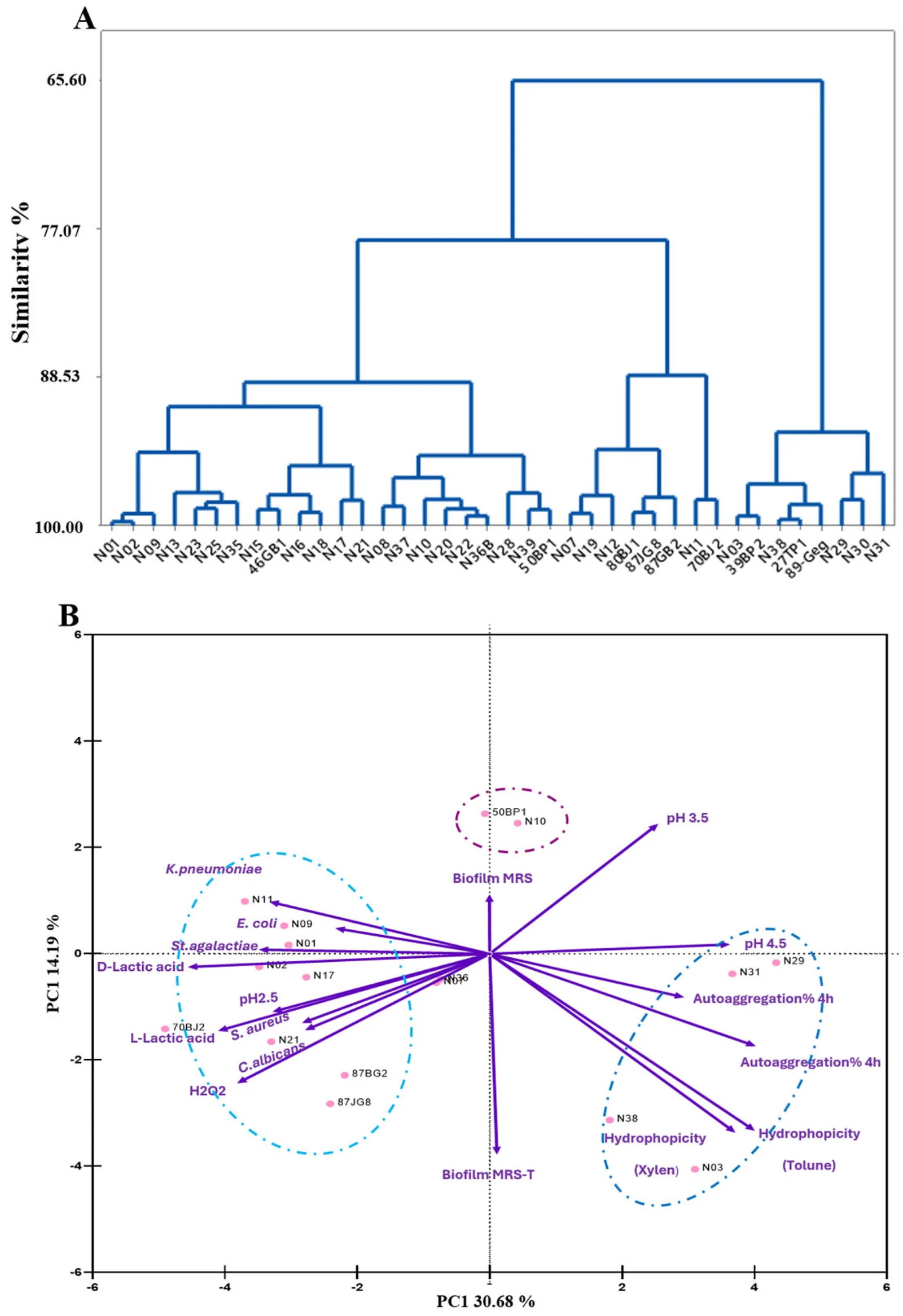
HCA and PCA of 38 selected vaginal LAB strains based on 17 probiotic-related variables, including antimicrobial activity against urogenital pathogens, production of lactic acid and H_2_O_2_, biofilm formation, cell surface hydrophobicity, auto-aggregation, and tolerance to acidic conditions. (**A**) Dendrogram generated by HCA using Euclidean distance, showing the clustering of LAB strains according to their phenotypic similarity, with similarity levels indicated up to 90%. (**B**) PCA biplot showing the distribution of the 17 variables projected onto the first two principal components (PC1 and PC2). Spatial proximity between variables or strains indicates similarity in functional profiles, whereas greater dispersion reflects dissimilarity. Smaller angles between vectors indicate positive correlations, larger angles indicate negative correlations, and angles close to 90° indicate no correlation. Circles highlight the variables contributing most strongly to the first two principal components and the strains most closely associated with them.

Hierarchical cluster analysis (HCA) and principal component analysis (PCA) were performed to evaluate the phenotypic relationships among the selected LAB strains based on the 17 probiotic-related variables evaluated in this work (Figure 8). The HCA dendrogram revealed a clear segregation of the isolates into two major clusters at approximately 65–70% similarity, highlighting the substantial functional diversity among the vaginal LAB strains (Figure 8A). One major cluster was mainly associated with antimicrobial-related characteristics, whereas the second cluster grouped strains displaying stronger adhesion- and colonization-associated properties. Several highly similar sub-clusters (>90% similarity) were also identified, including strain pairs or groups such as N01–N02, N23–N25–N35, N17–N21, and N29–N30–N31, suggesting closely related phenotypic profiles among these isolates.

PCA was subsequently applied to visualize the distribution of strains according to their functional characteristics (Figure 8B). Antibiotic susceptibility and hemolytic activity were excluded from this analysis because they represented qualitative variables and showed highly similar profiles among the strains (Supplementary Table S2).

The first two principal components explained 44.8% of the total variance, with PC1 and PC2 accounting for 30.6% and 14.1%, respectively. PC1 represented the main axis of functional differentiation and clearly separated antimicrobial-associated traits from adhesion-related properties. Variables linked to antimicrobial activity, including L- and D-lactic acid production, H_2_O_2_ production, and inhibitory activity against *E. coli*, *K. pneumoniae*, *S. aureus*, *St. agalactiae*, and *C. albicans*, were grouped on the negative side of PC1. Strains such as 70BJ2, N21B, 87BG2, and N17 showed the strongest association with this functional profile. In contrast, variables related to adhesion and persistence, including auto-aggregation (4 h and 24 h), cell surface hydrophobicity (xylene and toluene), and tolerance to acidic conditions (pH 4.5 and 3.5), were located on the positive side of PC1 and were mainly associated with strains N38, N03, N29, and N31.

PC2 further contributed to the discrimination of strains according to stress adaptation and biofilm-related characteristics. Negative PC2 loadings were mainly associated with hydrophobicity, H_2_O_2_ production, and biofilm formation under modified conditions (MRS-T), whereas positive loadings were related to acid tolerance and, to a lesser extent, biofilm formation in standard MRS medium. Interestingly, strains 50BP1 and N10 were positioned near the center of the PCA plot, indicating an intermediate phenotype combining moderate antimicrobial and adhesion-associated properties. Overall, both multivariate analyses demonstrated the marked phenotypic heterogeneity of the vaginal LAB isolates and allowed the identification of strains exhibiting distinct probiotic-associated functional profiles.

### 2.9. Functional Genomic Analysis of Lpb. plantarum N17 and 87JG8

Among the LAB with the highest capacities to inhibit the pathogens evaluated in this work were the strains N17 and 87JG8, both belonging to the species *Lpb. plantarum*. Although both strains were associated with antimicrobial-related traits, *Lpb. plantarum* N17 displayed a phenotype mainly linked to lactic acid production, whereas strain 87JG8 showed a stronger association with H_2_O_2_ production, suggesting distinct mechanisms involved in their potential protective effects against urogenital pathogens. Thus, a comparative genomic analysis between *Lpb.plantarum* N17 and strain 87JG8 could be valuable to identify the genetic determinants underlying their distinct antimicrobial-associated phenotypes, as well as to characterize whether these two strains possess other differences associated with their potential probiotic properties. For these reasons, the complete genome sequencing of both strains was obtained, and a comparative genomic analysis was performed.

For the genomic comparison, the type strain *Lpb.plantarum* DSM20174 was selected, as well as LAB originally isolated from the human vaginal tract that have been recognized as probiotic or potential probiotic strains (Supplementary Table S3). Then, *Lpb. plantarum* 87JG8 and N17 were compared with the strains DSM20174, ATG-K2, ATG-K6, ATG-K8, and SRCM100442 belonging to the same species, as well as with *Lacticaseibacillus (Lbs.) rhamnosus* GR-1, *Lactobacillus (Lb.) gasseri* DSM14869, *Lb. jensenii* SNUV360, *Lactococcus (Lc.) lactis* CV56, *Lmb. fermentum* NB-22, *Lmb. reuteri* RC-14, *Lb. crispatus* V4, CO3MRSI1, and CTV-05.

The draft genomes of N17 and 87JG8 consisted of 32 and 46 contigs, respectively (Table 3). Genome sizes of the two strains (3.26 Mb for N17 and 3.32 Mb for 87JG8) and their GC contents (44.4%) were highly similar to those of reference *Lpb. plantarum* strains, whose genome sizes ranged from 3.18 to 3.28 Mb and GC contents from 44.5 to 45.1% (Table 3). These genomic features further confirmed the taxonomic assignment of N17 and 87JG8 as members of the species *Lpb. plantarum*.

**Figure 9:**
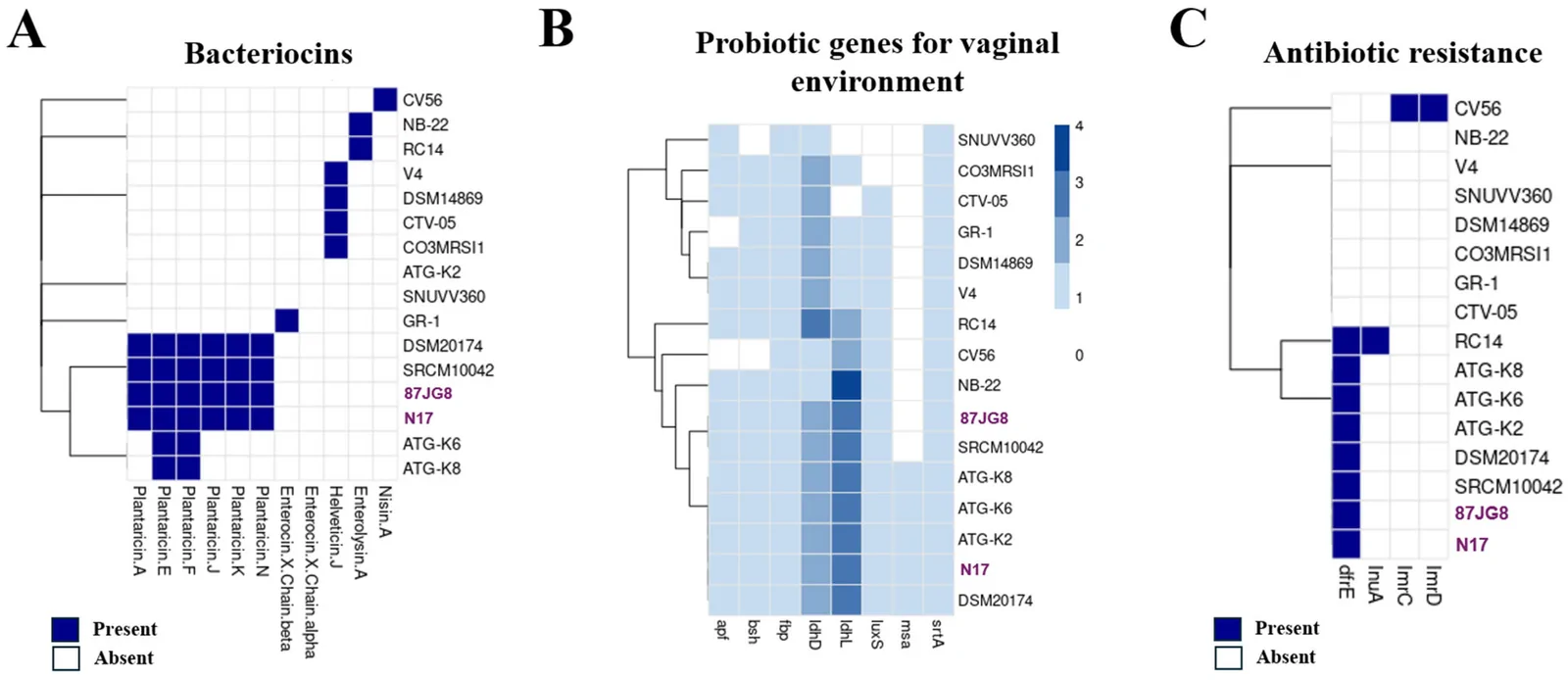
Genes in the vaginal isolates *Lpb. plantarum* N17 and 87JG8 genomes compared to genes present in the genomes of LAB originally isolated from the human vaginal tract that have been recognized as probiotic or potential probiotic strains. (**A**) Bacteriocins, (**B**) probiotic-related genes for the vaginal environment, and (**C**) antibiotic resistance genes. Colored boxes indicate the presence (blue) or absence (white) of genes. In addition, the number of homologous genes is depicted in different shades of blue.

Comparative genomic analysis revealed that the two strains, *Lpb. plantarum* N17 and 87JG8, shared a genomic repertoire highly similar to that of other *Lpb. plantarum* strains (Figure 9). Both strains harbored the complete set of plantaricin structural genes (plantaricin A, E, F, J, K and N), whereas enterolysin A, enterocin X, helveticin J, and nisin A genes were absent (Figure 9A). This bacteriocin profile was conserved among several of the *Lpb.plantarum* genomes analyzed and clearly distinguished them from the other vaginal lactobacilli included in the comparison. Of note, plantaricin genes were not detected in the genome of *Lpb. plantarum* ATG-K2, indicating differences among the strains of the same species.

Genes involved in host interaction and colonization, including the adhesion genes *apf* (aggregation-promoting factor), *fbp* (fibronectin-binding protein), *msa* (mannose-specific adhesin), and *srt*A (sortase A), as well as bile tolerance (*bsh*) and quorum sensing (*lux*S), were conserved among the *Lpb. plantarum* strains (Figure 9B). Of note, *Lpb. plantarum* 87JG8 and SRCM100442 do not possess the *msa* gene. In addition, the L-lactate dehydrogenase (*ldh*L) and D-lactate dehydrogenase (*ldh*D) genes were investigated in the genomes. Because *ldh*D and *ldh*L are represented by multiple homologs within the analyzed genomes, their distribution was evaluated based on gene copy number rather than simple presence or absence (Figure 9B). N17 and 87JG8 showed copy number profiles similar to those of reference *Lpb. plantarum* strains, whereas greater variability was observed among the non-*plantarum* species. The highest copy number for the ldhL gene was observed for *Lmb. fermentum* NB-22, a strain that had lower *ldh*D copy number compared to *Lpb. plantarum* strains.

A comparative analysis of safety-related genes was also performed by studying virulence and antimicrobial resistance determinants. The distribution of selected antimicrobial resistance genes was evaluated among the vaginal lactobacilli genomes (Figure 9C). Both *Lpb. plantarum* N17 and 87JG8 harbored the *drt*E and *lnu*A genes, a profile that was conserved across the *Lpb. plantarum* strains included in the analysis. The *drt*E gene has been associated with multidrug efflux systems, whereas *lnu*A encodes a lincosamide nucleotidyltransferase involved in resistance to lincosamide antibiotics. In contrast, the ABC transporter genes *lmr*C and *lmr*D, which have been implicated in multidrug efflux-mediated antibiotic resistance, were absent from N17, 87JG8, and the other *Lpb. plantarum* genomes, being detected only in *Lc. lactis* CV56. In addition, genome analysis revealed the absence of known virulence-associated genes in both N17 and 87JG8.

## 3. Discussion

The present study aimed to identify and characterize vaginal LAB with probiotic potential from a large collection of isolates obtained from Algerian women. Compared with previous studies describing a limited number of vaginal isolates, this work provides a broader functional characterization of vaginal LAB from an understudied population of North Africa. A large collection of isolates was initially obtained and subjected to a multistep screening strategy including antimicrobial activity, metabolite production, biofilm formation, aggregation capacity, hydrophobicity, acid tolerance, and safety evaluation. Moreover, the use of multivariate analyses such as HCA and PCA allowed the identification of distinct functional profiles among the strains, highlighting the marked strain-specific diversity that exists within the vaginal lactobacilli community. These results highlight the high functional diversity within vaginal microbiota and reinforce the concept that not all lactobacilli strains contribute equally to host protection, as previously reported [33].

The selection of potential probiotic strains for the vaginal tract performed here was based on the combination of two functional criteria: (i) broad-spectrum antimicrobial activity against Gram-positive and Gram-negative bacteria, as well as the yeast *C. albicans*, and (ii) the in vitro properties that allow the prediction of vaginal colonization and persistence. This multi-parameter selection strategy is critical, as antimicrobial activity alone does not guarantee probiotic efficacy. Instead, successful vaginal probiotics must integrate pathogen inhibition with colonization capacity, mediated by adhesion, aggregation, and biofilm formation. Then, from the 38 strains with the highest capacities to inhibit pathogens, a refined subset of 15 strains was selected based on superior performance across multiple functional traits, including: 10 *Lpb. plantarum*, 3 *Lmb. fermentum*, and 2 *Lgb. salivarius* strains. This stepwise selection approach reflects a rational pipeline for probiotic candidate identification, with translational potential.

Among the vaginal LAB strains evaluated, *Lmb. fermentum* 70BJ2 emerged as one of the most promising probiotic candidates due to its remarkable functional profile. This strain exhibited one of the strongest antimicrobial activities against all tested pathogens, which have been associated with aerobic vaginitis and vulvovaginal candidiasis [23]. These results are in line with previous studies demonstrating the abilities of vaginal LAB to inhibit pathogenic microorganisms. It was reported that lactobacilli isolated from healthy pregnant women have strong antimicrobial activities against *St. agalactiae*, *E. coli,* and *S. aureus* [21]. Similarly, [34] found that *Lb. delbrueckii* 45E exhibited remarkable inhibitory effects not only for *St. agalactiae*, *E. coli* and *Klebsiella* spp., but also for *Candida* spp. Our results also showed that *Lmb. fermentum* 70BJ2 was one of the strains with the highest ability for the production of H_2_O_2_ and D-lactic acid, two metabolites strongly associated with vaginal health and pathogen inhibition [20,35,36]. In addition, 70BJ2 showed excellent tolerance to acidic conditions, particularly at pH 2.5, suggesting a high capacity to survive harsh environmental conditions.

It was observed that a lactic acid-based vaginal gel is as effective as metronidazole for the treatment of bacterial vaginosis [37,38]. In vitro studies have shown that the incorporation of lactic acid at physiological concentrations exhibited a microbicidal effect against vaginosis-associated bacteria and *Candida* spp. without adversely affecting beneficial vaginal commensal bacteria [39,40]. Furthermore, lactic acid can also inactivate *Chlamydia trachomatis* [41], and the Human Immunodeficiency Virus [42]; and has been shown not only to exert direct antimicrobial effects but also to modulate host immune responses and enhance epithelial barrier integrity [43,44]. On the other hand, it has been demonstrated that H_2_O_2_ has a significant antimicrobial effect on vaginosis-associated bacteria, although supraphysiological concentrations are necessary to achieve this [39]. Furthermore, it was demonstrated that physiological concentrations of H_2_O_2_ are rapidly broken down by the vaginal fluid and mucosa [45]. However, studies suggested that the acidic environment generated by lactobacilli-derived lactic acid may potentiate the antimicrobial effects of H_2_O_2_ against vaginal pathogens [46]. Of note, the benefits of H_2_O_2_ in the vaginal mucosa can go beyond the direct antimicrobial effects since this molecule can stimulate epithelial cells to secrete antimicrobial metabolites and enhanced the activity of intrinsic protective factors, such as muramidase and lactoferrin, thereby strengthening the vaginal mucosal immune barrier [47]. Then, it is tempting to speculate that *Lmb. fermentum* 70BJ2 could contribute to vaginal health through multiple complementary mechanisms, including direct pathogen inhibition, reinforcement of the acidic vaginal environment, and enhancement of the local mucosal protective barrier. However, further in vitro and in vivo studies will be necessary to demonstrate these beneficial effects.

On the other hand, *Lpb. plantarum* N17 and 87JG8 displayed a broad inhibitory activity against urogenital pathogens evaluated in this work. Although both the N17 and 87JG8 strains were grouped within the antimicrobial-associated cluster in the PCA analysis, they exhibited distinct functional profiles. Strain N17 was more closely associated with elevated L-lactic acid production and displayed a more balanced phenotype linked to broad antimicrobial activity and adaptation to the vaginal environment. In contrast, strain 87JG8 showed a stronger association with H_2_O_2_ production and occupied a more extreme position within the antimicrobial cluster, suggesting a more specialized antimicrobial phenotype. These differences indicate that vaginal LAB strains may contribute to urogenital health through distinct mechanisms, even when sharing similar inhibitory activity against pathogens.

Another important difference between *Lpb.plantarum* N17 and 87JG8 is their capacities to form biofilms, being the 87JG8 the one with the most remarkable ability. Biofilm formation was identified as one of the key traits supporting vaginal colonization and persistence of LAB species on the vaginal epithelium [48]. Our findings confirmed that these properties are highly strain-dependent and influenced by environmental conditions, such as culture medium composition. The enhanced biofilm formation observed in MRS-T medium suggests that surface-active compounds (e.g., Tween 80) may interfere with adhesion, emphasizing the importance of experimental conditions when evaluating probiotic traits. Similarly, it was reported a high capacity for biofilm formation in most of the strains when cultured in media without Tween 80 [49,50]. Tween 80, a non-ionic surfactant, appears to interfere with biofilm development, whereas its removal enhances cell-substratum interaction. In the present study, the strongest biofilm formation among all tested strains was observed in *Lgb. salivarius* N03 and N38, followed by *Lpb. plantarum* 87JG8. In agreement with our findings, Ref. [51] found that *Lpb.plantarum* emerged as the strongest biofilm producer, likely due to its genomic versatility and adaptive capacity to diverse niches. However, our results showed that it is a strain-dependent property.

In agreement with our findings, previous studies described strain-dependent differences in the probiotic potential of vaginal LAB. For example, Ref. [52] evaluated fifteen vaginal *Lb. crispatus* strains for antimicrobial activity and found clear strain-specific differences in both the strength and spectrum of inhibition against several vaginal pathogens. To link phenotypic findings with genome sequences, we conducted comparative genomics studies using the strains N17 and 87JG8. The results revealed that both strains possess functional gene clusters, including those for bacteriocins, enzymes associated with lactic acid production, and adhesion factors similar to those found in other probiotic strains isolated from the vaginal tract. However, comparative genomics did not identify any substantial differences between *Lpb. plantarum* N17 and 87JG8. These findings underscore that genomic similarity does not necessarily predict functional similarity, highlighting the importance of integrating comparative genomics with phenotypic characterization when selecting potential probiotic strains. Even within the same species, antimicrobial capacity and metabolite production can differ substantially, supporting the need for rigorous screening strategies such as the one applied in this study.

The safety profile of the selected strains further supports their suitability as probiotic candidates. Phenotypic analyses demonstrated that all vaginal LAB isolates were susceptible to clinically relevant antibiotics, in agreement with the safety criteria established for lactobacilli [53,54]. The limited resistance observed for vancomycin and aminoglycosides is consistent with the intrinsic resistance mechanisms widely described for lactobacilli [53,55]. Importantly, these resistance traits are generally considered non-transferable because they are not associated with mobile genetic elements, representing a negligible risk of horizontal gene transfer [55,56]. Comparative genomic analyses further confirmed the favorable safety profile of the selected strains, revealing the absence of known virulence-associated genes and the presence of only a limited repertoire of antimicrobial resistance determinants commonly found in other probiotic *Lactiplantibacillus* strains. In addition, none of the vaginal LAB isolates exhibited β-hemolytic activity, providing further evidence of their safety for potential probiotic applications.

## 4. Materials and Methods

### 4.1. Sampling Population

The study population consisted of 160 premenopausal Algerian women who attended the Mother and Child Specialized Hospital (EHS) Mariem Bouatoura, Batna, Algeria, between December 2022 and January 2024. During routine preventive tests for cervical cancer, examinations were performed using a vaginal speculum. Vaginal exudates were collected from the lateral wall of the vagina using two sterile cotton swabs, one for culture and isolation, and the other for preparing a fresh mount and Gram-stained slides. The speculum was also used to perform the Fische test and measure vaginal pH. All participants included in the present study were aged between 19 and 45 years and had not used any probiotics, antibiotics, antifungal compounds, vaginal medications, suppositories, or contraceptive spermicides in the previous 30 days of the consultation. Furthermore, they had not engaged in sexual intercourse one week before sample collection. Each woman was asked to complete a questionnaire that included several questions related to health (Supplementary Table S4). The study was conducted in accordance with ethical principles and was approved by the Institutional Ethics Committee of the University of Mohamed Khider, Biskra, Algeria (Approval No. 002/2026), as well as by the Mother and Child Specialized Hospital (EHS) Mariem Bouatoura, Batna, Algeria (Approval No. 1909/2022).

### 4.2. Isolation of Vaginal LAB Strains

The 160 samples were divided into two groups. For the first group, 80 swabs were placed directly into 5 mL of MRS broth medium (Liofilchem, Roseto degli Abruzz, Italy) and incubated at 37 °C for 24 to 48 h under anaerobic conditions with 5% CO_2_ using a NUVE EC160 CO_2_ incubator. Following incubation, the samples were streaked on MRS agar (Liofilchem, Roseto degli Abruzz, Italy) and incubated again for an additional 48 h. For the second group, the remaining 80 swabs were vortexed for 30 s in physiological saline solution. The resulting suspension was serially diluted, and 100 μL of each dilution was plated on MRS agar plates in triplicate. The plates were then incubated under the same conditions as described in the first group. After incubation, individual colonies were randomly selected, purified on MRS broth/agar, and examined based on their morphology (size, shape, color, pigment nature, boundary, elevation, and texture). Colonies that were identified as rod-shaped or short, oval-shaped, and confirmed as Gram-positive with negative catalase and indole tests were selected as vaginal LAB strains and stored on MRS broth containing 40% glycerol at −20 °C for further experiments.

### 4.3. Evaluation of Antimicrobial Activity

The indicator strains used in this study included *St. agalactiae*, *E. coli*, *K. pneumoniae*, and *C. albicans*. All pathogens were isolated from women with urogenital infections in private microbiological laboratories in Biskra and Batna, Algeria. The identification of *Enterobacteriaceae* and *C. albicans* was performed using the API 20E and API Candida systems (bioMérieux, Marcy-l’Etoile, France), respectively. *St. agalactiae* was identified using the VITEK 2 system (bioMérieux, Marcy-l’Etoile, France), followed by aggregation testing with the BIO-RAD Pastorex Strep Kit (Marnes-la-Coquette, France) for confirmation of group B streptococci. The reference strain *S. aureus* ATCC 25923 was also used in the studies. The antibiotic susceptibility profiles of *K. pneumoniae*, *E. coli*, and *St. agalactiae* were determined using standard antimicrobial susceptibility testing procedures. The susceptibility patterns, including inhibition zone diameters and/or minimum inhibitory concentration (MIC) values, were interpreted according to the guidelines of the Clinical and Laboratory Standards Institute (CLSI) and/or the European Committee on Antimicrobial Susceptibility Testing (EUCAST). The complete results are presented in Supplementary Table S5.

All clinical isolates were tested for antibiotic susceptibility according to EUCAST guidelines. Strains were stored in Brain Heart Infusion (BHI) broth (Liofilchem, Roseto degli Abruzz, Italy) supplemented with 20% glycerol at −20 °C.

The antimicrobial activity of vaginal LAB isolates was tested using the agar spot-diffusion method as described by [57] against the five pathogenic microorganisms. Briefly, LAB strains were subcultured three times in 5 mL of MRS broth at 37 °C under 5% CO_2_ for 18 h. After the final culture, LAB was washed twice with sterile 0.9% NaCl saline solution and adjusted to a turbidity equivalent to the 0.5 McFarland standard. An aliquot (5 µL) of each suspension was spotted onto an MRS agar plate. The plates were subsequently incubated at 37 °C under 5% CO_2_ for 24 h. Following incubation, the plates were overlaid with 10 mL of BHI soft agar (0.7%) previously inoculated with pathogenic bacteria or fungi at a final concentration of 10^8^ CFU/mL. Finally, the plates were incubated aerobically at 37 °C for an additional 24 h. The diameters of the inhibition zones surrounding the LAB spots were measured using vernier calipers and recorded in millimeters. All assays were performed in triplicate.

### 4.4. Production of D-/L-Lactic Acid

The D- and L-lactic acid concentrations in culture-free supernatants (CFS) were measured based on colorimetric determination using an L and D lactate dehydrogenase kit (Megazyme International Ireland Ltd., Co. Wicklow, Ireland). According to the manufacturer’s instructions, ten milliliters of overnight-grown cultures of each vaginal LAB strain were centrifuged at 10,000 rpm for 10 min at 4 °C to obtain CFS. Then, 100 µL of supernatant and 900 µL of distilled water were added to a test tube and mixed well to achieve D- and L- lactic acid concentrations ranging from 0.3 to 3 g/L. The assays were performed in triplicate.

### 4.5. Production of Hydrogen Peroxide

The H_2_O_2_ production was evaluated using a quantitative method based on the reaction between the OxiRed probe and H_2_O_2_ in the presence of horseradish peroxidase (HRP), which produces a colored product. This assay was performed using the commercial kit BioVision’s Hydrogen Peroxide Assay Kit (BioVision Incorporated, South Milpitas, CA, USA). Before the assay, the vaginal LAB strains were activated in MRS broth and incubated at 37 °C for 12 h. The CFS of each strain was obtained by centrifugation for 15 min at 1000× *g* at 4 °C. In a 96-well plate, 02–50 µL of supernatant was added to each well in triplicate. A total of 50 µL of reaction mix, containing 46 µL of assay buffer, 2 µL of OxiRed probe (Abcam, Cambridge, United Kingdom), and 2 µL of HRP solution, was added to each sample and the H_2_O_2_ standard. The mixture was then mixed thoroughly and incubated at room temperature for 10 min. The H_2_O_2_ production of the vaginal LAB strains and the standard curve were measured at λmax = 570 nm using a microplate reader (Buenos Aires, Argentina).

### 4.6. Evaluation of Biofilm Formation

The biofilm formation of vaginal LAB strains was tested according to the method described by [58], using two different culture media: MRS and MRS without Tween 80 (MRS-T). Bacterial pellets were obtained from the third subculture in both media by centrifugation at 4000 rpm for 15 min and washing twice with sterile saline solution. Then, bacteria were resuspended in the same solution to a final concentration of 2 × 10^8^ CFU/mL. A volume of 200 µL of the bacterial suspension was inoculated into 5 mL of each broth medium and was vortexed well. Subsequently, an aliquot of 200 µL of the suspension was added to sterile U-bottom 96-well polystyrene microplates and incubated for 72 h in a 5% CO_2_ incubator at 37 °C. After the incubation, the contents of the wells were gently removed, and the wells were washed with phosphate-buffered saline (PBS). Adherent biofilms were stained for 30 min with 200 µL of 0.1% crystal violet (CV) in an isopropanol-methanol-PBS solution (1:1:18, *v*/*v*). The wells were then rinsed twice with 200 µL of distilled water per well to remove excess stain and air-dried. To solubilize CV bound to the adherent biofilm, 200 µL of 30% glacial acetic acid was added to each well.

Biofilm quantification was carried out by measuring optical density (OD) at 560 nm using a SPECTROstra Nano microplate reader (BMG LABTECH, Ortenberg, Germany). Additionally, a sterile culture medium was always included as a negative control.

The cut-off OD (ODc) was determined as the mean OD of the negative control plus three SD. Based on the ODc value, LAB strains’ s biofilm formation was classified as follows: OD ≤ ODc, non-adherent; ODc < OD ≤ 2 × ODc, weak biofilm producer; 2 × ODc < OD ≤ 4 × ODc, moderate biofilm producer; 4 × ODc < OD ≤ 8 × ODc, strong biofilm producer; OD > 8 × ODc, very strong biofilm producer.

### 4.7. Auto-Aggregation and Hydrophobicity Tests

The auto-aggregation abilities of vaginal LAB strains were assessed according to the method described by [59,60] with some modification. Briefly, the bacterial strains were subcultured three times in MRS broth. The cells were then harvested by centrifugation at 4000 rpm for 15 min, washed twice with 0.9% NaCl saline solution, and resuspended in 10 mL of the same solution. The suspensions were adjusted to an OD of 0.600 ± 0.02 at 600 nm (A_0_) and then vortexed for 10 s. The tubes were incubated at 37 °C for 4 h and 24 h. After incubation, a 2 mL aliquot from the upper part of the suspension was carefully collected and transferred to a glass cuvette to measure absorbance at 600 nm (A_t_) using a spectrophotometer (spectro: J P selecta UV-2005).

The percentage of auto-aggregation was expressed as: Auto-aggregation (%) = [1 − (A_t_/A_0_)] × 100. In this formula, A_0_= absorbance at 0 h (initial) and A_t_ = absorbance at time t (4 and 24 h).

Cell surface hydrophobicity was assessed using the bacterial adhesion to hydrocarbons method [58]. This method is based on the ability of LAB to be captured into a hydrocarbon phase (xylene or toluene) from a 0.9% NaCl saline solution. The initial OD of the preparation of cell suspension was measured as in the auto-aggregation assay at 600 nm (A_0_). Then, 0.6 mL of xylene/toluene was added to 3 mL the bacterial suspension the mixture was vortexed for 1 min and incubated at room temperature for 15 min to allow phases separation. After incubation, the OD of the aqueous phase was measured (A_1_).

The hydrophobicity percentage was calculated using the following formula: Hydrophobicity (%) = [(A_0_ − A_1_)/A_0_] × 100. In this formula, A_0_ = absorbance at 0 h (initial) and A_1_ = absorbance after phase separation.

### 4.8. Tolerance to Low pH

The tolerance of LAB strains to acidic conditions was assessed at different pH levels (6.5, 4.5, 3.5, and 2.5), following the method described by [61] with minor modifications. Briefly, LAB strains were subcultured three times, then centrifuged and washed twice with sterile saline solution. The bacterial suspensions were then adjusted to a concentration of 2 × 10^8^ CFU/mL. Aliquots of 10 µL from each strain suspension were inoculated into 200 µL of MRS broth previously adjusted to the respective pH values and dispensed into a sterile 96-well microplate. The microplate was incubated anaerobically at 37 °C for 3 h and 24 h using a CO_2_-generating anaerobic system (GENbox CO_2_, (bioMérieux, Marcy-l’Etoile—France)). After incubation, and spreading serial dilutions, viable cells count was performed by cultivation on MRS agar. The percentage of survival rate at low pH was calculated using the following formula:

Survival rate % = (Growth at test pH (CFU/mL)/Growth at control pH (CFU/mL) × 100

### 4.9. Evaluation of Antibiotic Susceptibility

The antibiotic susceptibility of 38 selected vaginal LAB was performed using the disk diffusion technique on MRS agar plates against 17 antibiotics as follows: ampicillin (AMP; 10 µg), kanamycin (K; 30 µg), amikacin (AK; 30 µg), ciprofloxacin (CIP; 5 µg), erythromycin (E; 15 µg), cefotaxime (CTX; 30 µg), vancomycin (VA; 30 µg), amoxicillin/clavulanic acid (AMC; 30 µg), chloramphenicol (C; 30 µg), clindamycin (DA; 2 µg), gentamicin (CN; 120 µg), streptomycin (S; 10 µg), tetracycline (TE; µg), azithromycin (AZM; 15 µg), doxycycline (DO; 30 µg), and rifampicin (RIF; 5 µg). Individual colonies from 48 h fresh culture were adjusted in a 5 mL tube to 0.5 McFarland (1 × 10^8^ CFU/mL); a 100 µL aliquot of the suspension was then swabbed onto MRS agar. The disks were placed on the agar plates and incubated at 37 °C for 48 h under anaerobic conditions using a CO_2_-generating anaerobic system (Genbag CO_2_). *E. coli* ATCC8739 was used as a control strain for antibiotics susceptibility testing. The results were categorized as resistance (R), intermediate (I), or susceptible (S) based on the interpretive zone diameters outlined in the antimicrobial disk susceptibility test standards [31,32].

### 4.10. Hemolysis Test

Hemolytic activity was assessed by streaking strains onto Columbia blood agar containing 7% defibrinated sheep blood. After 48 h of incubation at 37 °C under anaerobic condition using a CO_2_-generating anaerobic system (Genbag CO_2_), plates were examined for α-hemolysis (green-hued zones around colonies), β-hemolysis (clear zones around colonies), and γ-hemolysis (no hemolysis). The vaginal *St. agalactiae* ASM and *S. aureus* ATCC 25923 were used as positive controls, while *Enterococcus (E.) faecium* Fm k89 was used as negative control.

### 4.11. Genome and 16s ARN Sequencing

PCR amplification was performed using the PrimeSTAR HS DNA Polymerase kit (Takara Bio Inc., Shiga, Japan). For colony PCR, a single LAB colony from 15 strains was picked using a sterile pipette tip and directly used as the DNA template in a 50 μL reaction mixture containing 10 μL of 5× PrimeSTAR Buffer (Mg^2+^ plus), 4 μL of dNTP mixture (2.5 mM each), 10–15 pmol of each forward and reverse primer, 0.5 μL of PrimeSTAR HS DNA Polymerase (2.5 U/μL), and sterile distilled water up to the final volume. All reactions were prepared on ice according to the manufacturer’s instructions. PCR amplification was carried out using a three-step cycling protocol consisting of initial denaturation at 98 °C for 10 s, followed by 30 cycles of denaturation at 98 °C for 10 s, annealing at 55 °C for 5–15 s, and extension at 72 °C for 1 min/kb. In selected reactions, a two-step PCR protocol (98 °C for 10 s and 68 °C for 1 min/kb) was also applied.

PCR products were analyzed by agarose gel electrophoresis, where DNA fragments were separated on an agarose gel containing Midori Green DNA stain incorporated prior to polymerization and visualized under UV illumination using HyperLadder 1 kb as a molecular weight marker. PCR products of the expected size were purified using the NucleoSpin^®^ Gel and PCR Clean-up kit (Macherey-Nagel, Westphalia, Germany) according to the manufacturer’s instructions.

The concentration and purity of purified DNA were assessed using a NanoDrop One spectrophotometer (Thermo Scientific, Waltham, MA, USA), and absorbance ratios (A260/A280 and A260/A230) were used to evaluate DNA quality.

For Sanger sequencing, purified PCR products were prepared by mixing 1 μL of purified DNA with 0.19 μL of primer (50 pmol/μL) and 19.81 μL of nuclease-free water to obtain a final volume of 21 μL. Samples were submitted for sequencing after proper mixing and quality verification.

### 4.12. Complete Genome Sequencing and Bioinformatic Analysis

Genomic DNA was extracted from *Lpb. plantarum* N17 and 87JG8 using the EasyPure^®^ Bacteria Genomic DNA Kit (TransGen Biotech Co., Ltd., Beijing, China) following the manufacturer’s instructions. DNA concentration was measured using a Nabi spectrophotometer (Micro Digital, Seoul, Korea), and DNA integrity was assessed by electrophoresis on a 1% agarose gel.

Whole-genome sequencing was performed on an Illumina MiSeq platform (Illumina Inc., San Diego, CA, USA) using 2 × 150 bp paired-end reads. Sequencing libraries were prepared with the TruSeq DNA HT Sample Preparation Kit (Illumina Inc.) according to the manufacturer’s recommendations. Raw reads were quality-filtered and trimmed using PrinSeq-lite v0.20.4 (minimum mean quality score of 20 and minimum read length of 70 bp).

Genomic sequence data for different bacterial strains were obtained from the National Center for Biotechnology Information (NCBI) database. Reference genomes were retrieved from GenBank and annotated using the Rapid Annotation using Subsystem Technology (RAST) server. Protein sequences corresponding to the genes of interest were obtained from the Kyoto Encyclopedia of Genes and Genomes (KEGG) database and used as queries for BLASTP searches against the annotated genomes within the RAST platform.

For analysis of L-lactate dehydrogenase (*ldh*L) and D-lactate dehydrogenase (*ldh*D) genes, the predicted protein FASTA files for each strain were downloaded from the NCBI database and analyzed using the dbCAN3 server. The distribution of genes among the analyzed genomes was visualized by heatmap generation using the R package pheatmap (version 1.0.12). The search for bacteriocin-encoding genes was performed using the Bagel 4 Server and the blastp algorithm. Virulence genes were investigated using the VFDB Database. All the procedures were performed as described previously [62].

### 4.13. Statistical Analysis

Statistical analyses were performed using GraphPad Prism v 9.5.1 software. Data were analyzed by one-way or two-way ANOVA tests, followed by Tukey test multiple comparison between different samples or Dunnett’s multiple comparison test when compared with controls, as well as a two-tailed *t*-test. The data are reported as the mean of three repetitions ± standard deviation; *p*-values < 0.05 were defined as statistically significant. The histograms and biplot principal component analyses (PCA) were generated in GraphPad Prism v 9.5.1 software, while correlation matrices and dendrograms were generated in Minitab v20.

## 5. Conclusions

This study demonstrates that a stepwise, multi-criteria selection strategy is essential for identifying high-potential vaginal probiotic candidates aimed at protecting the vaginal mucosa against pathogens. Starting from 259 isolates, the progressive reduction to 38 and ultimately to 15 strains allowed the identification of bacteria combining antimicrobial activity, metabolic functionality, adhesion capacity, and safety. Notably, the strains *Lmb. fermentum* 70BJ2, *Lpb. plantarum* N17 and *Lpb. plantarum* 87JG8 emerged as promising probiotic candidates for the vaginal tract. The present work provides the scientific rationale to perform further ex vivo and in vivo studies to confirm their probiotic efficacy and their potential in promoting vaginal health.

Beyond the identification of novel probiotic candidates, this work emphasizes the importance of investigating the vaginal microbiota of geographically underrepresented populations. The isolation of functionally promising lactobacilli from healthy Algerian women demonstrates that North African populations constitute a valuable and largely unexplored reservoir of probiotic diversity. Expanding microbiome research to these populations will not only improve our understanding of vaginal microbial ecology but may also facilitate the discovery of region-specific probiotic strains with unique functional properties for the prevention and management of urogenital infections.

## Figures and Tables

**Table 1:** Identification of vaginal LAB strains by 16S rRNA gene sequencing analysis.

Strains	Characteristics	Identification by 16S rRNA
N1	Short bacilli, Gram+, Catalase−	*Lactiplantibacillus plantarum*
N2	Short bacilli, Gram+, Catalase−	*Lactiplantibacillus plantarum*
N3	Short bacilli, Gram+, Catalase−	*Ligilactobacillus salivarius*
N7	Short bacilli, Gram+, Catalase−	*Lactiplantibacillus plantarum*
N9	Short bacilli, Gram+, Catalase−	*Limosilactobacillus fermentum*
N11	Short bacilli, Gram+, Catalase−	*Lactiplantibacillus plantarum*
N17	Short bacilli, Gram+, Catalase−	*Lactiplantibacillus plantarum*
N21	Short bacilli, Gram+, Catalase−	*Lactiplantibacillus plantarum*
N31	Short bacilli, Gram+, Catalase−	*Limosilactobacillus fermentum*
N36	Short bacilli, Gram+, Catalase−	*Lactiplantibacillus plantarum*
N38	Short bacilli, Gram+, Catalase−	*Ligilactobacillus salivarius*
50Bp1	Short bacilli, Gram+, Catalase−	*Lactiplantibacillus plantarum*
70BJ2	Short bacilli, Gram+, Catalase−	*Limosilactobacillus fermentum*
87JG8	Short bacilli, Gram+, Catalase−	*Lactiplantibacillus plantarum*
87BG2	Short bacilli, Gram+, Catalase−	*Lactiplantibacillus plantarum*

**Table 2:** Survival rate (%) of vaginal LAB in low pH conditions. Data are expressed as mean ± standard deviation (SD) of three independent experiments.

Strains	Survival Rate at pH 4.5 (After 24 h)	Survival Rate at pH 3.5 (After 24 h)	Survival Rate at pH 2.5 (After 3 h)
N1	97.6± 0.03	06.7 ± 0.2	24.6 ± 0.8
N2	89.0 ± 0.05	04.6 ± 0.9	31.9 ± 0.7
N3	99.0± 0.09	07.6 ± 0.8	14.6 ± 0.9
N7	98.9 ± 0.03	23.9 ± 0.9	52.4 ± 0.7
N9	100.6 ± 0.07	10.2 ± 0.4	39.7 ± 0.9
N11	80.0 ± 0.07	15.9 ± 0.5	85.6 ± 1.0
N17	88.8 ± 0.03	20.3 ± 0.5	19.9 ± 0.3
N21	84.9 ± 0.03	11.5 ± 0.7	29.3 ± 0.2
N31	104.5 ± 0.07	41.5 ± 0.8	10.7 ± 0.2
N36	103.1 ± 0.08	11.0 ± 0.5	26.6 ± 0.7
N38	112.1 ± 0.02	05.0 ± 0.3	15.0 ± 1.0
50Bp1	89.8± 0.02	43.7 ± 0.7	19.6 ± 0.7
70BJ2	94.9 ± 0.01	08.5 ± 0.9	91.8 ± 0.7
87BG2	82.3 ± 0.02	12.0 ± 0.2	55.9 ± 0.2
87JG8	87.8 ± 0.02	06.9 ± 0.5	92.7 ± 0.8

**Table 3:** General genomic features of LAB isolated from the human vaginal tract that have been recognized as probiotic or potential probiotic strains.

Strain	Genome Size	GC Content	Number of Contigs
DSM20174	3,250,154	44.5	1
ATG-K2	3,175,098	45.1	1
ATG-K6	3,262,505	44.5	1
ATG-K8	3,275,764	44.5	1
SRCM100442	3,223,643	44.6	1
87JG8	3,318,729	44.4	46
N17	3,259,985	44.4	32
GR-1	2,968,535	46.6	1
V4	2,091,889	37.0	252
CO3MRSI1	2,345,902	37.1	1
CTV-05	2,364,583	37.1	25
DSM14869	1,951,552	35.0	1
SNUV360	1,672,949	34.4	1
CV56	2,518,737	35.1	1
NB-22	2,011,311	51.8	137
RC-14	2,011,203	38.6	95

## Data Availability

The original contributions presented in the study are included in the article; further inquiries can be directed to the corresponding authors.

## Supplementary Materials

The following supporting information can be downloaded at: https://www.mdpi.com/article/10.3390/antibiotics15090875/s1. Supplementary Figure S1. Growth inhibition (mm) by live vaginal LAB strains against (A) *E. coli*, (B) *K. pneumoniae*. (C) Representative images of inhibition zones on agar plates. Bars represent mean values, and error bars indicate the standard deviations (SD) of triplicate inhibition zone measurements. One-way ANOVA (*p* < 0.05 and 95% CI) was applied for single pathogens, followed by Tukey’s post hoc test for comparison across pathogen pairs. Supplementary Figure S2. Growth inhibition (mm) by live vaginal LAB strains against (A) *S. aureus* and (B) *St. agalactiae*. (C) Representative image of inhibition zone on agar plates. Bars represent mean values, and error bars indicate the standard deviations (SD) of triplicate inhibition zone measurements. One-way ANOVA (*p* < 0.05 and 95% CI) was applied for single pathogen, followed by Tukey’s post hoc test for comparison across pathogen pairs. Supplementary Figure S3. Growth inhibition (mm) by live vaginal LAB strains against (A) *C. albicans*. (B) Representative image of inhibition zone on agar plates. Bars represent mean values, and error bars indicate the standard deviations (SD) of triplicate inhibition zone measurements. One-way ANOVA (*p* < 0.05 and 95% CI) was applied for single pathogen followed by Tukey’s post hoc test was applied for comparison across pathogen pairs. Supplementary Figure S4. (A) L-lactate, D-lactate; (B) hydrogen peroxide production by individual vaginal LAB strains. Bars represent mean values, and error bars indicate the standard deviations (SD) of triplicate measurements. One-way ANOVA (*p* < 0.05 and 95%CI) was applied for single metabolite analyses, followed by Dunnett’s post hoc test against the positive control strains (*L. acidophilus* ATCC 4356). Supplementary Figure S5. Biofilm formation by vaginal LAB strains in MRS (A) and MRS-T (B); bars represent mean values, and error bars indicate the SD from triplicate experiments; lines indicate the respective cut-off values. Two-way ANOVA with *p* < 0.05 and a 95% confidence interval was used, followed by a Tukey test for multiple comparisons between the groups. Supplementary Figure S6. Auto-aggregation abilities of vaginal LAB strains at 4 h (A) and 24 h (b). Bars represent mean values, and error bars indicate the SD from triplicate experiments. Two-way ANOVA (*p* < 0.05 and 95% CI) was used to evaluate the combined effects of time × LAB strains on auto-aggregation. Followed by Tukey’s post hoc test for multiple pairwise comparisons among factors. Supplementary Figure S7. Cell surface hydrophobicity to (A) xylene and (B) toluene abilities of vaginal LAB strains. Bars represent mean values, and error bars indicate the SD from triplicate experiments. Two-way ANOVA (*p* < 0.05 and 95%CI) was used to evaluate the combined hydrophobic solvent × LAB strains on hydrophobicity ability. Followed by Tukey’s post hoc test for multiple pairwise comparisons among factors. Supplementary Table S1. Survival rate (%) of vaginal LAB in low pH conditions. Supplementary Table S2. Correlation coefficient of the 17 probiotic characteristics of 38 vaginal LAB strains with the first 5PCs. Supplementary Table S3. General information on the LAB strains used for genomic comparison. Supplementary Table S4. Questionnaire on genital infections in sampled women. Supplementary Table S5. Antibiotic susceptibility profile of *K. pneumoniae*, *E. coli*, and *St. agalactiae*.

## References

[1] Samama M., Ueno J., Carvalho De Arruda Veiga E., Piscopo R.C.C.P., Ikeda F., Pires De Lemos N., Tadeu Bidinotto L., Guimarães Da Silva M., Jarmy Di Bella Z., Entezami F. (2025). Vaginal Microbiome and Its Relationship with Assisted Reproduction: A Systematic Review and Meta-Analysis. Life.

[2] Zhu B., Tao Z., Edupuganti L., Serrano M.G., Buck G.A. (2022). Roles of the Microbiota of the Female Reproductive Tract in Gynecological and Reproductive Health. Microbiol. Mol. Biol. Rev..

[3] Shen L., Zhang W., Yuan Y., Zhu W., Shang A. (2022). Vaginal Microecological Characteristics of Women in Different Physiological and Pathological Period. Front. Cell. Infect. Microbiol..

[4] Condori-Catachura S., Ahannach S., Ticlla M., Kenfack J., Livo E., Anukam K.C., Pinedo-Cancino V., Collado M.C., Dominguez-Bello M.G., Miller C. (2025). Diversity in Women and Their Vaginal Microbiota. Trends Microbiol..

[5] Morsli M., Gimenez E., Magnan C., Salipante F., Huberlant S., Letouzey V., Lavigne J.-P. (2024). The Association between Lifestyle Factors and the Composition of the Vaginal Microbiota: A Review. Eur. J. Clin. Microbiol. Infect. Dis..

[6] Borgdorff H., Van Der Veer C., Van Houdt R., Alberts C.J., De Vries H.J., Bruisten S.M., Snijder M.B., Prins M., Geerlings S.E., Schim Van Der Loeff M.F. (2017). The Association between Ethnicity and Vaginal Microbiota Composition in Amsterdam, the Netherlands. PLoS ONE.

[7] Ncib K., Bahia W., Leban N., Mahdhi A., Trifa F., Mzoughi R., Haddad A., Jabeur C., Donders G. (2022). Microbial Diversity and Pathogenic Properties of Microbiota Associated with Aerobic Vaginitis in Women with Recurrent Pregnancy Loss. Diagnostics.

[8] Ouarabi L., Drider D., Taminiau B., Daube G., Bendali F., Lucau-Danila A. (2021). Vaginal Microbiota: Age Dynamic and Ethnic Particularities of Algerian Women. Microb. Ecol..

[9] Jbara S., Idrissi A.A., Fadel S., Al Idrissi N., Rhalem W., Allali I., Bakri Y., Tissir R., Ghazal H., Ezziyyani M., Kacprzyk J., Balas V.E. (2024). 16S rRNA Gene-Amplicon-Based Profiling of the Vaginal Microbiome From North African Women. International Conference on Advanced Intelligent Systems for Sustainable Development (AI2SD’2023).

[10] Occhipinti S., Incognito G.G., Palumbo M. (2024). The Influence of the Vaginal Ecosystem on Vaginitis, Bacterial Vaginosis, and Sexually Transmitted Diseases: An Epidemiological Study and Literature Review. Arch. Gynecol. Obstet..

[11] Zhou Z., Feng Y., Xie L., Ma S., Cai Z., Ma Y. (2024). Alterations in Gut and Genital Microbiota Associated with Gynecological Diseases: A Systematic Review and Meta-Analysis. Reprod. Biol. Endocrinol..

[12] Borrego-Ruiz A., Borrego J.J. (2025). Microbial Pathogens Linked to Vaginal Microbiome Dysbiosis and Therapeutic Tools for Their Treatment. Acta Microbiol. Hell..

[13] La Rosa R., Johansen H.K., Molin S. (2022). Persistent Bacterial Infections, Antibiotic Treatment Failure, and Microbial Adaptive Evolution. Antibiotics.

[14] Boahen A., Than L.T.L., Loke Y.-L., Chew S.Y. (2022). The Antibiofilm Role of Biotics Family in Vaginal Fungal Infections. Front. Microbiol..

[15] Muzny C.A., Sobel J.D. (2022). The Role of Antimicrobial Resistance in Refractory and Recurrent Bacterial Vaginosis and Current Recommendations for Treatment. Antibiotics.

[16] Ravel J., Gajer P., Abdo Z., Schneider G.M., Koenig S.S.K., McCulle S.L., Karlebach S., Gorle R., Russell J., Tacket C.O. (2011). Vaginal Microbiome of Reproductive-Age Women. Proc. Natl. Acad. Sci. USA.

[17] Wang X., Jiang Q., Tian X., Chen W., Mai J., Lin G., Huo Y., Zheng H., Yan D., Wang X. (2025). Metagenomic Analysis Reveals the Novel Role of Vaginal Lactobacillus Iners in Chinese Healthy Pregnant Women. npj Biofilms Microbiomes.

[18] Asadi A., Lohrasbi V., Abdi M., Mirkalantari S., Esghaei M., Kashanian M., Oshaghi M., Talebi M. (2022). The Probiotic Properties and Potential of Vaginal Lactobacillus Spp. Isolated from Healthy Women against Some Vaginal Pathogens. Lett. Appl. Microbiol..

[19] Shazadi K., Arshad N. (2022). Evaluation of Inhibitory and Probiotic Properties of Lactic Acid Bacteria Isolated from Vaginal Microflora. Folia Microbiol..

[20] Pino A., Bartolo E., Caggia C., Cianci A., Randazzo C.L. (2019). Detection of Vaginal Lactobacilli as Probiotic Candidates. Sci. Rep..

[21] Kumherová M., Veselá K., Kosová M., Mašata J., Horáčková Š., Šmidrkal J. (2021). Novel Potential Probiotic Lactobacilli for Prevention and Treatment of Vulvovaginal Infections. Probiot. Antimicrob. Proteins.

[22] Tachedjian G., Aldunate M., Bradshaw C.S., Cone R.A. (2017). The Role of Lactic Acid Production by Probiotic Lactobacillus Species in Vaginal Health. Res. Microbiol..

[23] Avitabile E., Menotti L., Croatti V., Giordani B., Parolin C., Vitali B. (2024). Protective Mechanisms of Vaginal Lactobacilli against Sexually Transmitted Viral Infections. Int. J. Mol. Sci..

[24] Monti F., Giordani B., Fedi S., Ghezzi D., Galletti P., Mercolini L., Mandrioli R., Parolin C., Luppi B., Vitali B. (2025). Vaginal Lactobacillus Gasseri Biosurfactant: A Novel Bio- and Eco-Compatible Anti-Candida Agent. Biofilm.

[25] Leccese Terraf M.C., Juárez Tomás M.S., Rault L., Le Loir Y., Even S., Nader-Macías M.E.F. (2016). Biofilms of Vaginal Lactobacillus Reuteri CRL 1324 and Lactobacillus Rhamnosus CRL 1332: Kinetics of Formation and Matrix Characterization. Arch. Microbiol..

[26] Chantanawilas P., Pahumunto N., Teanpaisan R. (2024). Aggregation and Adhesion Ability of Various Probiotic Strains and Candida Species: An in Vitro Study. J. Dent. Sci..

[27] Zuñiga Vinueza A.M. (2024). Probiotics for the Prevention of Vaginal Infections: A Systematic Review. Cureus.

[28] Lehtoranta L., Ala-Jaakkola R., Laitila A., Maukonen J. (2022). Healthy Vaginal Microbiota and Influence of Probiotics Across the Female Life Span. Front. Microbiol..

[29] Mashatan N., Heidari R., Altafi M., Amiri A., Ommati M.M., Hashemzaei M. (2023). Probiotics in Vaginal Health. Pathog. Dis..

[30] Dillen J., Dricot C.E.M.K., Croatti V., Lebeer S. (2026). The Female Urogenital Microbiome: Ecological Insights, Therapeutic Strategies, and Molecular Mechanisms. Cell Host Microbe.

[31] Charteris W.P., Kelly P.M., Morelli L., Collins J.K. (1998). Antibiotic Susceptibility of Potentially Probiotic Lactobacillus Species. J. Food Prot..

[32] Melo T.A., Dos Santos T.F., Pereira L.R., Passos H.M., Rezende R.P., Romano C.C. (2017). Functional Profile Evaluation of *Lactobacillus Fermentum* TCUESC01: A New Potential Probiotic Strain Isolated during Cocoa Fermentation. BioMed Res. Int..

[33] Petrova M.I., Reid G., Vaneechoutte M., Lebeer S. (2017). Lactobacillus Iners: Friend or Foe?. Trends Microbiol..

[34] Bnfaga A.A., Lee K.W., Than L.T.L., Amin-Nordin S. (2023). Antimicrobial and Immunoregulatory Effects of Lactobacillus Delbrueckii 45E against Genitourinary Pathogens. J. Biomed. Sci..

[35] Tyssen D., Wang Y.-Y., Hayward J.A., Agius P.A., DeLong K., Aldunate M., Ravel J., Moench T.R., Cone R.A., Tachedjian G. (2018). Anti-HIV-1 Activity of Lactic Acid in Human Cervicovaginal Fluid. mSphere.

[36] Happel A.-U., Kullin B., Gamieldien H., Wentzel N., Zauchenberger C.Z., Jaspan H.B., Dabee S., Barnabas S.L., Jaumdally S.Z., Dietrich J. (2020). Exploring Potential of Vaginal Lactobacillus Isolates from South African Women for Enhancing Treatment for Bacterial Vaginosis. PLoS Pathog..

[37] Campaner A.B., Rosário Sica A.C.A., D’ Avila Curi F.S., Marchetti G., Dias G.S., Teixeira B.L. (2025). In Vivo Assessment of the Effect of Gel Containing Lactic Acid and Glycogen on Vaginal Microbiota and pH of Asymptomatic Women of Reproductive Age. PLoS ONE.

[38] Tidbury F., Brülhart G., Müller G., Pavicic E., Weidlinger S., Eichner G., Von Wolff M., Stute P. (2025). Effectiveness and Tolerability of Lactic Acid Vaginal Gel Compared to Oral Metronidazole in the Treatment of Acute Symptomatic Bacterial Vaginosis: A Multicenter, Randomized-Controlled, Head-to-Head Pilot Study. BMC Women’s Health.

[39] O’Hanlon D.E., Moench T.R., Cone R.A. (2011). In Vaginal Fluid, Bacteria Associated with Bacterial Vaginosis Can Be Suppressed with Lactic Acid but Not Hydrogen Peroxide. BMC Infect. Dis..

[40] Paniágua A.L., Correia A.F., Pereira L.C., De Alencar B.M., Silva F.B.A., Almeida R.M., De Medeiros Nóbrega Y.K. (2021). Inhibitory Effects of Lactobacillus Casei Shirota against Both Candida Auris and Candida Spp. Isolates That Cause Vulvovaginal Candidiasis and Are Resistant to Antifungals. BMC Complement. Med. Ther..

[41] Gong Z., Luna Y., Yu P., Fan H. (2014). Lactobacilli Inactivate Chlamydia Trachomatis through Lactic Acid but Not H_2_O_2_. PLoS ONE.

[42] Aldunate M., Tyssen D., Johnson A., Zakir T., Sonza S., Moench T., Cone R., Tachedjian G. (2013). Vaginal Concentrations of Lactic Acid Potently Inactivate HIV. J. Antimicrob. Chemother..

[43] Delgado-Diaz D.J., Jesaveluk B., Hayward J.A., Tyssen D., Alsatian A., Potgieter M., Bell L., Ross E., Iranzadeh A., Allali I. (2022). Lactic Acid from Vaginal Microbiota Enhances Cervicovaginal Epithelial Barrier Integrity by Promoting Tight Junction Protein Expression. Microbiome.

[44] Nazarova N.M., Devyatkina A.R., Mezhevitinova E.A., Prilepskaya V.N., Alieva L.E., Sycheva E.G., Muravieva V.V. (2025). Lactic Acid as a Key Factor in Restoring Vaginal Microbiota: Physiological and Clinical Aspects. Obstet. Gynecol..

[45] Miko E., Barakonyi A. (2023). The Role of Hydrogen-Peroxide (H_2_O_2_) Produced by Vaginal Microbiota in Female Reproductive Health. Antioxidants.

[46] Atassi F., Servin A.L. (2010). Individual and Co-Operative Roles of Lactic Acid and Hydrogen Peroxide in the Killing Activity of Enteric Strain Lactobacillus Johnsonii NCC933 and Vaginal Strain Lactobacillus Gasseri KS120.1 against Enteric, Uropathogenic and Vaginosis-Associated Pathog: Hydrogen Peroxide and Lactic Acid Co-Operate for Killing. FEMS Microbiol. Lett..

[47] Sgibnev A.V., Kremleva E.A. (2015). Vaginal Protection by H_2_O_2_-Producing Lactobacilli. Jundishapur J. Microbiol..

[48] Xu Z., Premarathna M., Li Y., Yin X., Soteyome T., Liu J., Seneviratne G. (2025). Current Knowledge on the Polymicrobial Interaction and Biofilm between Saccharomyces and Lactobacillaceae: Regulatory Mechanisms and Applications. Biofilm.

[49] Silva J.A., Marchesi A., Aristimuño Ficosecco M.C., Nader-Macías M.E.F. (2022). Functional and Safety Characterization of Beneficial Vaginal Lactic Acid Bacteria for the Design of Vaginal Hygiene Products. J. Appl. Microbiol..

[50] Miranda M.H., Nader M.E.F. (2020). Advances in the Design of A Multi-Strain Homologous Probiotic Formula for Cattle.

[51] Parolin C., Croatti V., Laghi L., Giordani B., Tondi M.R., De Gregorio P.R., Foschi C., Vitali B. (2021). Lactobacillus Biofilms Influence Anti-Candida Activity. Front. Microbiol..

[52] Argentini C., Fontana F., Alessandri G., Lugli G.A., Mancabelli L., Ossiprandi M.C., Van Sinderen D., Ventura M., Milani C., Turroni F. (2022). Evaluation of Modulatory Activities of Lactobacillus Crispatus Strains in the Context of the Vaginal Microbiota. Microbiol. Spectr..

[53] Anisimova E.A., Yarullina D.R. (2019). Antibiotic Resistance of LACTOBACILLUS Strains. Curr. Microbiol..

[54] Li C., Cheng Y., Zong T., Guo S., Zhu H., Dai P., Chen C., Wang H. (2026). Alleviative Effect of Vaginal Lactobacilli with Probiotic Potential from Healthy Chinese Women on Bacterial Vaginosis Caused by Gardnerella Vaginalis in Mice. Probiot. Antimicrob. Proteins.

[55] Nøhr-Meldgaard K., Struve C., Ingmer H., Koza A., Al-Nakeeb K., Agersø Y. (2023). Antimicrobial Susceptibility Testing and Tentative Epidemiological Cut-off Values for Lactobacillaceae Family Species Intended for Ingestion. Front. Antibiot..

[56] Campedelli I., Mathur H., Salvetti E., Clarke S., Rea M.C., Torriani S., Ross R.P., Hill C., O’Toole P.W. (2019). Genus-Wide Assessment of Antibiotic Resistance in *Lactobacillus* Spp.. Appl. Environ. Microbiol..

[57] Scillato M., Spitale A., Mongelli G., Privitera G.F., Mangano K., Cianci A., Stefani S., Santagati M. (2021). Antimicrobial Properties of *Lactobacillus* Cell-free Supernatants against Multidrug-resistant Urogenital Pathogens. MicrobiologyOpen.

[58] Uezen J.D., Ficoseco C.A., Nader-Macías M.E.F., Vignolo G.M. (2023). Identification and Characterization of Potential Probiotic Lactic Acid Bacteria Isolated from Pig Feces at Various Production Stages. Can. J. Vet. Res..

[59] Ahire J.J., Sahoo S., Kashikar M.S., Heerekar A., Lakshmi S.G., Madempudi R.S. (2023). In Vitro Assessment of Lactobacillus Crispatus UBLCp01, Lactobacillus Gasseri UBLG36, and Lactobacillus Johnsonii UBLJ01 as a Potential Vaginal Probiotic Candidate. Probiot. Antimicrob. Proteins.

[60] Kos B., Šušković J., Vuković S., Šimpraga M., Frece J., Matošić S. (2003). Adhesion and Aggregation Ability of Probiotic Strain *Lactobacillus Acidophilus* M92: Adhesion of *L. acidophilus* M92. J. Appl. Microbiol..

[61] Boubakri K., Melian C., Cataldo P.G., Hebert E.M., Idoui T., Vignolo G. (2025). Exploring the Functional and Probiotic Potential of Bacteriocin-Producing Lactobacilli from Algerian Kaddid. Biocatal. Agric. Biotechnol..

[62] Elean M., Arroyo Guerra A., Albarracin L., Nishiyama K., Kitazawa H., Audisio M.C., Villena J. (2025). Genomic Characterization of the Honeybee–Probiotic Strain *Ligilactobacillus salivarius* A3iob. Animals.

