# Supplementary material for: Selection and Characterization of Vaginal Lactobacilli from Healthy Algerian Women with Potential Probiotic Properties Against Urogenital Pathogens
Source: Antibiotics (Basel). 2026 Sep 7;15(9):875. doi: 10.3390/antibiotics15090875 (PMC13603449; doi:10.3390/antibiotics15090875)
Supplement: Supplementary file 1 [file antibiotics-15-00875-s001.zip › antibiotics-4509063-supplementary.pdf]

Supplementary Figures and Tables.

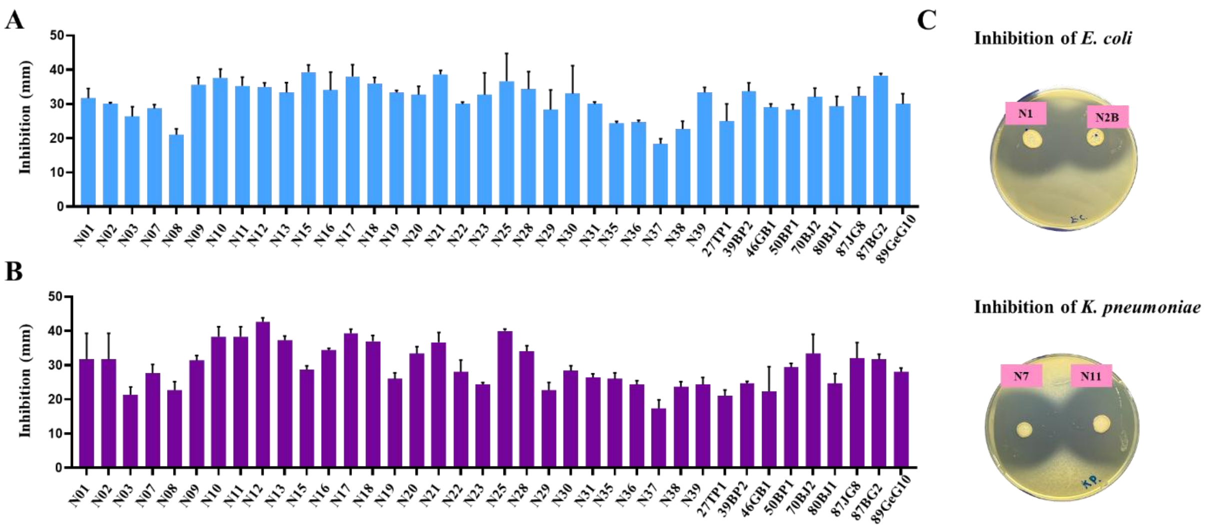

Supplementary Figure S1. Growth inhibition (mm) by live vaginal LAB strains against (A) *E. coli*, (B) *K. pneumoniae*. (C) Representative images of inhibition zone on agar plates. Bars represent mean values, and error bars indicate the standard deviations (SD) of triplicate inhibition zone measurement. One-way ANOVA ($P<0.05$ and 95 % CI) was applied for single pathogen followed by *Tukey's post hoc* test was applied for comparison across pathogen pairs.

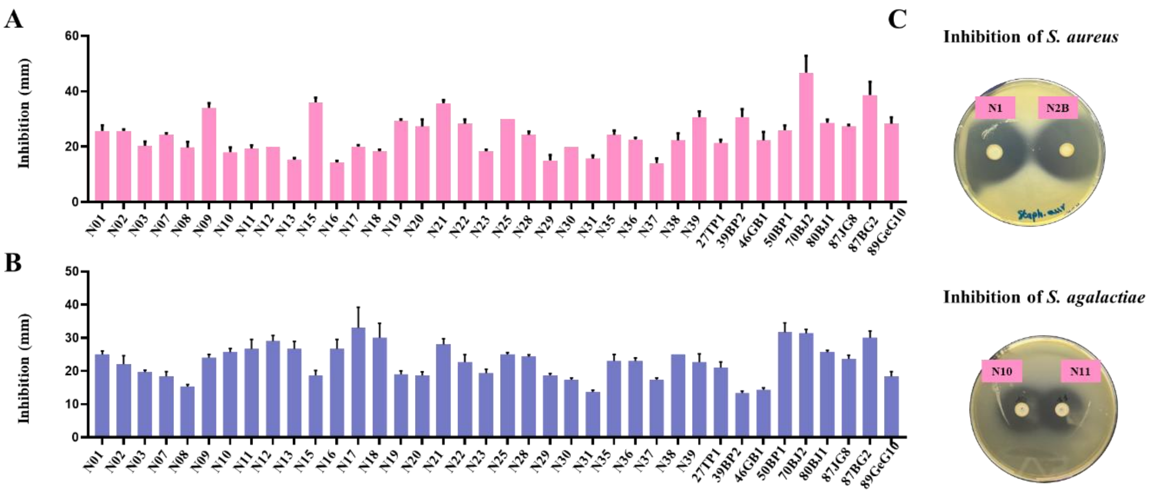

Supplementary Figure S2. Growth inhibition (mm) by live vaginal LAB strains against (A) *S. aureus*, (B) *St. agalactiae.* (C) Representative image of inhibition zone on agar plates, Bars represent mean values, and error bars indicate the standard deviations (SD) of triplicate inhibition zone measurement. One-way ANOVA ($P<0.05$ and 95 % CI) was applied for single pathogen followed by *Tukey's post hoc* test was applied for comparison across pathogen pairs.

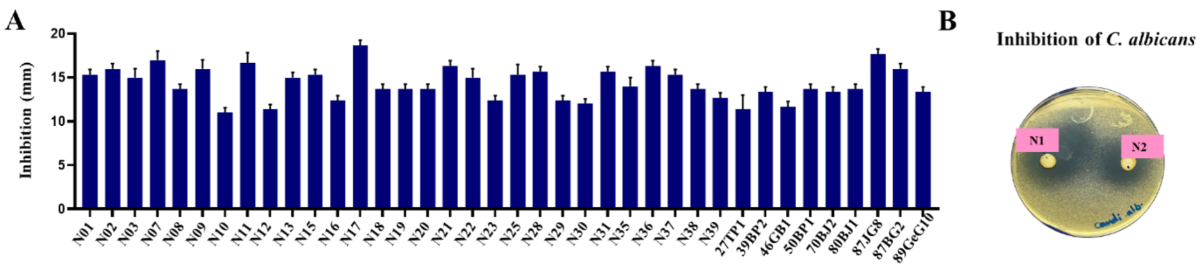

Supplementary Figure S3. Growth inhibition (mm) by live vaginal LAB strains against (A) *C. albicans*. (B) Representative image of inhibition zone on agar plates, Bars represent mean values, and error bars indicate the standard deviations (SD) of triplicate inhibition zone measurement. One-way ANOVA (P<0.05 and 95 % CI) was applied for single pathogen followed by *Tukey's post hoc* test was applied for comparison across pathogen pairs.

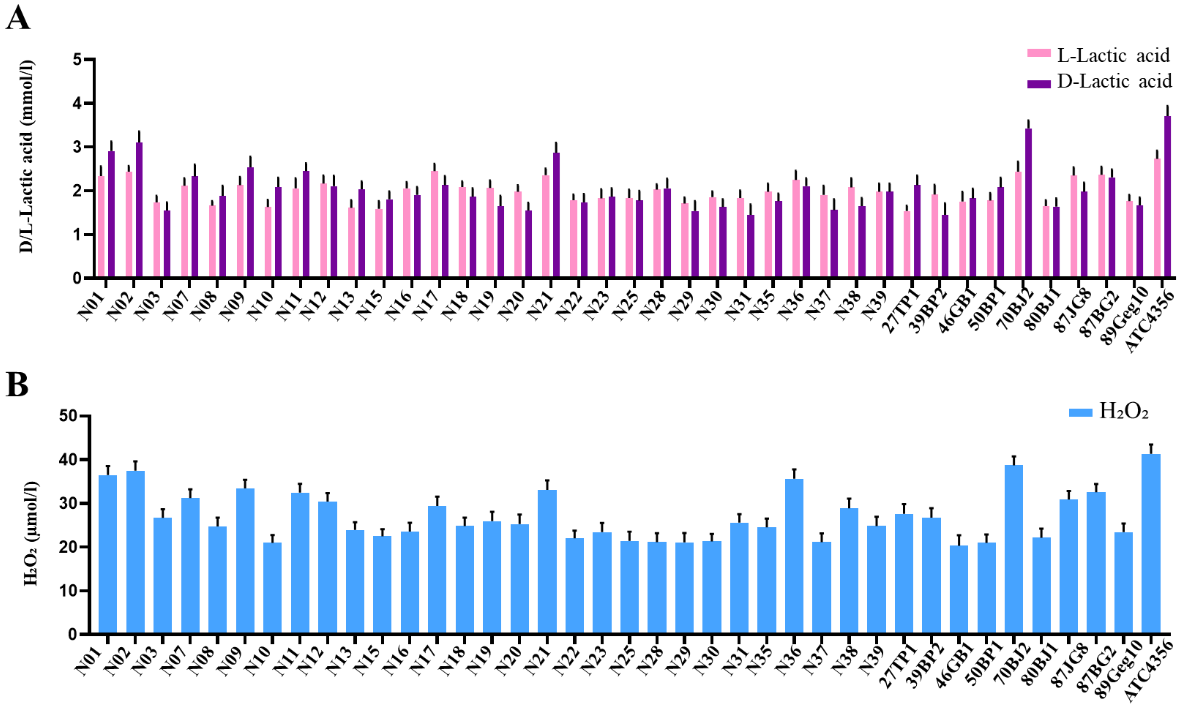

Supplementary Figure S4. (A) L-lactate, D-lactate, (B) hydrogen peroxide production by individual vaginal LAB strains. Bars represent mean values, and error bars indicate the standard deviations (SD) of triplicate measurement. One-way ANOVA (P<0.05 and 95 % CI) was applied for single metabolite analyses, followed by *Dunnett's post hoc* test against the positive control strains (*Lb. acidophilus* ATCC 4356). And two-way ANOVA ($p < 0.05$, 95% confidence interval) was used to analyse L- and D-lactic acid production, followed by Tukey's multiple comparison test.

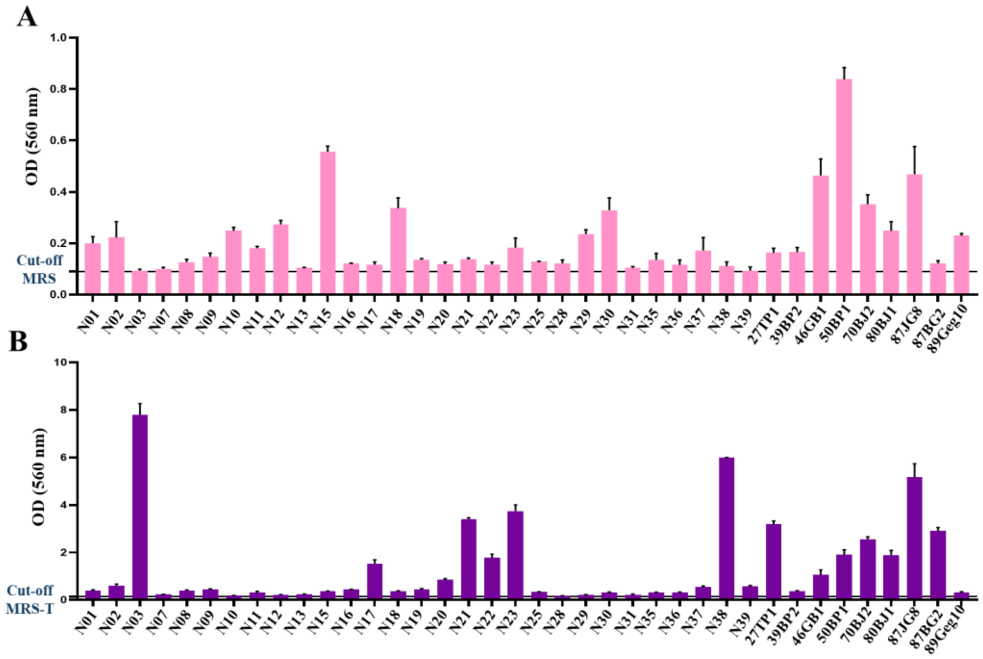

Supplementary Figure S5. Biofilm formation by vaginal LAB strains in MRS (A) and MRS-T (B), bars represent mean value, and error bars indicate the SD from triplicate experiments; lines indicate the respective cut-off values. Two-way ANOVA with $P<0.05$ and 95 % confidence interval was used, when it followed by *Tukey* test multiple comparison between the groups.

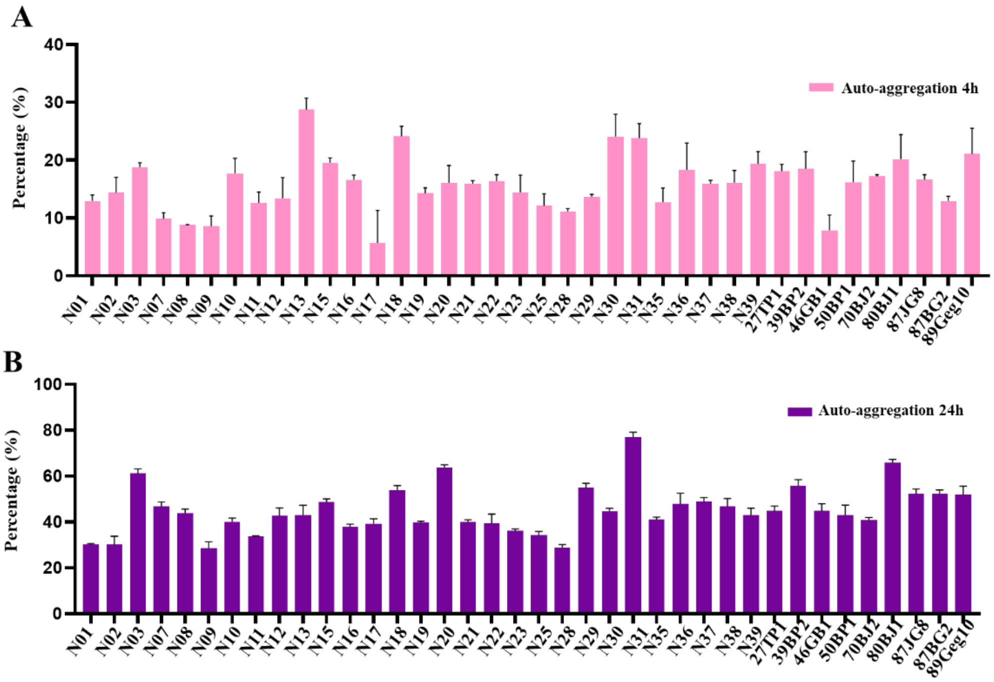

Supplementary Figure S6. Auto-aggregation abilities of vaginal LAB strains at 4h (A) and 24h (b). Bars represent mean value, and error bars indicate the SD from triplicate experiments. Tow way- ANOVA (*P*<0.05 and 95%CI) was used to evaluate the combined effects times × LAB strains on auto-aggregation. Followed by *Tukey's* post hoc test for multiple pairwise comparison among factors.

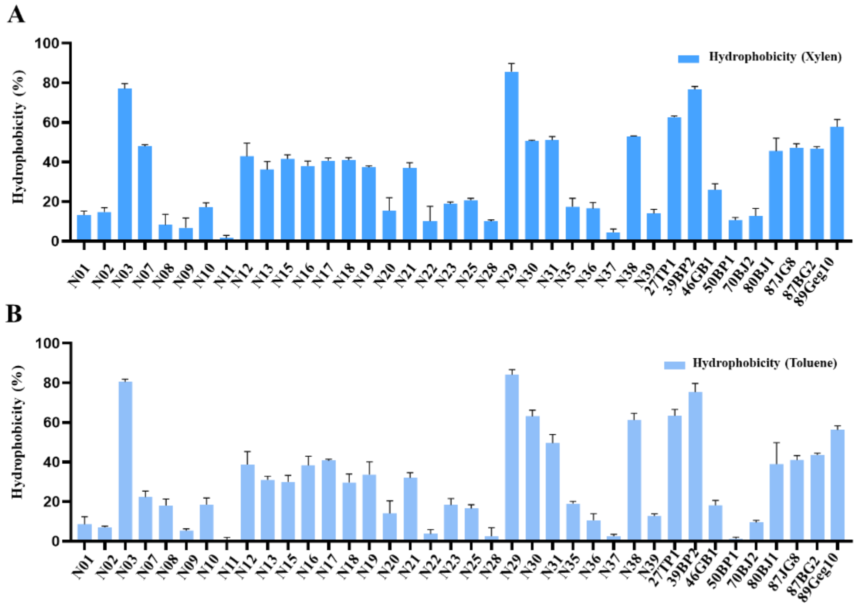

Supplementary Figure S7. Cell surface hydrophobicity to (A) xylene and (B) toluene abilities of vaginal LAB strains. bars represent mean value, and error bars indicate the SD from triplicate experiments, Tow way- ANOVA (P<0.05 and 95 % CI) was used to evaluate the combined hydrophobic solvent× LAB strains on hydrophobicity ability. Followed by *Tukey's* post hoc test for multiple pairwise comparison among factors.

Supplementary Table S1. Survival rate (%) of vaginal LAB in low pH conditions

Strains	Survival rate (%) after 24 h		Survival rate (%) after 3h
	pH 4.5	pH 3.5	pH2.5
**N1**	97.6± 0.03	6.7 ± 0.2	24.6 ± 0.8
**N2**	89.0 ± 0.05	4.6 ± 0.9	31.9 ± 0.7
**N3**	99.0± 0.09	7.6 ± 0.8	14.6 ± 0.9
**N7**	98.9 ± 0.03	23.9 ± 0.9	52.4 ± 0.7
**N8**	95.3 ± 0.05	25.6 ± 0.3	23.4 ± 0.9
**N9**	100.6 ± 0.07	10.2 ± 0.4	39.7 ± 0.9
**N10**	97.2± 0.08	26.2 ± 0.2	23.0 ± 0.2
**N11**	80.0 ± 0.07	15.9 ± 0.5	85.6 ± 1.0
**N12**	100.4 ± 0.06	45.2 ± 0.9	65.2 ± 0.4
**N13**	96.7 ± 0.04	11.1 ± 0.7	46.4 ± 0.9
**N15**	110.5 ± 0.1	37.1 ± 0.7	20.4 ± 0.2
**N16**	112.4 ± 0.05	33.2 ± 0.9	7.0 ± 1.0
**N17**	88.8 ± 0.03	20.3 ± 0.5	19.9 ± 0.3
**N18**	105.6 ± 0.04	32.0 ± 0.9	15.3 ± 0.8
**N19**	91.2 ± 0.06	23.2 ± 0.8	46.2 ± 0.7
**N20**	104.6 ± 0.03	16.2 ± 0.9	27.5 ± 0.2
**N21**	84.9 ± 0.03	11.5 ± 0.7	29.3 ± 0.2
**N22**	94.1± 0.08	15.6 ± 0.8	27.4 ± 0.5
**N23**	100.0 ± 0.07	5.6 ± 0.7	44.7 ± 0.2
**N25**	92.5 ± 0.06	4.1 ± 0.2	42.5 ± 1.0
**N28**	68.0 ± 0.2	27.0 ± 0.7	19.4 ± 0.9
**N29**	109.2 ± 0.06	45.3 ± 0.6	12.4 ± 1.0
**N30**	105.5 ± 0.07	36.8 ± 0.8	11.5 ± 1.0
**N31**	104.5 ± 0.07	41.5 ± 0.8	10.7 ± 0.2
**N35B**	74.4 ± 0.09	0.8 ± 0.5	29.1 ± 0.5
**N36B**	103.1 ± 0.08	11.0 ± 0.5	26.6 ± 0.7
**N37**	109.6± 0.08	42.7 ± 0.7	22.7 ± 0.9
**N38**	112.1 ± 0.02	5.0 ± 0.3	15. ± 1.
**N39**	98.1 ± 0.08	38.2 ± 0.9	28.4 ± 0.8
**27TP1**	95.4 ± 0.03	8.5 ± 0.7	15.2 ± 0.5
**39Bp2**	84.7 ± 0.02	3.7 ± 0.5	16.1 ± 0.5
**46BG1**	98.2 ± 0.06	15.5 ± 0.8	12.3 ± 0.3
**50Bp1**	89.8± 0.02	43.7 ± 0.7	19.6 ± 0.7
**70BJ2**	94.9 ± 0.01	8.5 ± 0.9	91.8 ± 0.7
**80BJ1**	87.0 ± 0.02	9.1 ± 0.5	71.0 ± 0.7
**87BG2**	82.3 ± 0.02	12. ± 0.2	55.9 ± 0.2

**87JG8**	87.8 ± 0.02	6.9 ± 0.5	92.7 ± 0.8
**89Geg10**	113.7 ± 0.05	12.4 ± 0.7	23.9 ± 0.8

Data are expressed as mean ± standard deviation (SD) of three independent experiments.

Supplementary Table S2. Correlation coefficient of the 17 probiotic characteristics of 38 vaginal LAB strains with the first 5 PCs.

Variable	PC1	PC2
E. coli inhibition	-0.42725	0.109873893
K. pneumoniae inhibition	-0.60956	0.223651776
S. aureus inhibition	-0.51057	-0.276114291
St. agalactiae inhibition	-0.63878	0.018009256
C. albicans inhibition	-0.51927	-0.250414755
L-Lactic acid production	-0.75127	-0.278959105
D-Lactic acid production	-0.83452	-0.047669156
H_2O_2 production	-0.69833	-0.46854844
Biofilm MRS	-0.00057	0.255511238
Biofilm MRS-T	0.016199	-0.725613012
Autoaggregation % 4 h	0.433085	-0.157332689
Autoaggregation % 24 h	0.59224	-0.333892648
Hydrophopicity in xylene %	0.546816	-0.646760702
Hydrophopicity in tolune %	0.590347	-0.640186618
Tolerance at pH 4.5	0.535223	0.040361898
Tolerance at pH 3.5	0.375048	0.55693619
Tolerance at pH 2.5	-0.60086	-0.209874134
Eigen value	5.215	2,413
Proportion	30.68%	14.19%
Cumulative	30.68%	44.87%

Supplementary Table S3. General information of the LAB strains used for genomic comparison.

Strain	Species	GenBank assembly	NCBI RefSeq assembly	Status
DSM20174	*Lactiplantibacillus plantarum*	GCA_014131735.1	GCF_014131735.1	Complete
ATG-K2	*Lactiplantibacillus plantarum*	GCA_003597635.1	GCF_003597635.1	Complete
ATG-K6	*Lactiplantibacillus plantarum*	GCA_003597595.1	GCF_003597595.1	Complete
ATG-K8	*Lactiplantibacillus plantarum*	GCA_003597615.1	GCF_003597615.1	Complete
SRCM100442	*Lactiplantibacillus plantarum*	GCA_009913655.1	GCF_009913655.1	Complete
87JG8	*Lactiplantibacillus plantarum*			Contigs
N17	*Lactiplantibacillus plantarum*			Contigs
GR-1	*Lacticaseibacillus rhamnosus*	GCA_024665595.1	GCF_024665595.1	Complete
V4	*Lactobacillus crispatus*	GCA_004681235.1	GCF_004681235.1	Contigs
CO3MRSI1	*Lactobacillus crispatus*	GCA_003795065.1	GCF_003795065.1	Complete
CTV-05	*Lactobacillus crispatus*	GCA_000165885.1	-	Scaffold
DSM14869	*Lactobacillus gasseri*	GCA_002287905.1	GCF_002287905.1	Complete
SNUV360	*Lactobacillus jensenii*	GCA_001936235.1	GCF_001936235.1	Complete
CV56	*Lactococcus lactis*	GCA_000192705.1	GCF_000192705.1	Complete
NB-22	*Limosilactobacillus fermentum*	GCA_000496435.1	GCF_000496435.1	Scaffold
RC-14	*Limosilactobacillus reuteri*	GCA_002762415.1	GCF_002762415.1	Contig

Supplementary Table S4. Questionnaire on genital infections in sampled women.

Code: ……… **First name:** …………… **Last name:** ……………
Date of birth (dd/mm/yyyy): //___ **Age:** ___ years ___ months
Weight: ☐ low ☐ average ☐ obese Numerical value: ……………
Residence: ……………
Hospitalized: ☐ yes ☐ no If yes, department: ……………
Family situation: ☐ married ☐ single ☐ divorced If married/divorced, since when? ……………
Number of children: ……………
Pregnancy: ☐ yes ☐ no If yes, gestational age: ……………
Delivery method: ☐ natural (vaginal) ☐ cesarean
Previous pregnancy history: ……………
Menopausal status: ☐ menopausal ☐ premenopausal
Contraceptive method: ☐ IUD / spermicides ☐ oral pills ☐ other: …………
Menstruation: ……………
History of urinary tract infection: ☐ first infection ☐ recurrent
History of vaginal infection: ☐ first infection ☐ recurrent
Associated Symptoms

1. Itching (pruritus) ☐ yes ☐ no Start date: //___
2. Burning sensation ☐ yes ☐ no Start date: //___
3. Vaginal discharge ☐ yes ☐ no Start date: //___
4. Irritation ☐ yes ☐ no Start date: //___
5. Pain during intercourse ☐ yes ☐ no Start date: //___
6. Inflammation (vagina/vulva/cervix) ☐ yes ☐ no Start date: //___

Characteristics of Vaginal Discharge
Color: ☐ white/clear ☐ whitish ☐ grayish ☐ greenish
Amount: ☐ abundant ☐ moderate ☐ low
Odor: ☐ odorless ☐ foul-smelling (fishy)
Other: ……………
Use of tampons: ☐ yes ☐ no
Sexual Activity
Coitus: ☐ yes ☐ no
Frequency: ☐ normal (2–3 times/week) ☐ abnormal
Hygiene & Lifestyle
Bath additives: ☐ yes ☐ no Genital washing: ☐ yes ☐ no Tight/synthetic clothing: ……………
Chronic Diseases
☐ anemia ☐ diabetes ☐ hypertension ☐ heart disease ☐ liver disease ☐ kidney disease ☐ lung disease ☐ asthma ☐ cancer ☐ HIV/immune deficiency ☐ other: ……………
Current Treatment
Under treatment: ☐ yes ☐ no Medications used: ……………
Antibiotic Treatment
Currently taking antibiotics: ☐ yes ☐ no

Hormone therapy used: …………… Antibiotics used: ……………

Antibiotics active against: ☐ yeast ☐ bacteria ☐ both

Amount taken: ☐ full course ☐ partial ☐ none

Method: ☐ oral ☐ local Dose: ……… times/day Duration: ……………

Effectiveness: ▫ / ☐ very effective ▫ / ☐ effective ▫ / ☐ not much

Self-Medication

Self-medication: ☐ yes ☐ no

If yes:

Medication: Name: …………… Most effective: …………… Method: …………… Dose: ……… times/day Duration: ……………

Herbal therapy: Herb name: …………… Most effective: …………… Method: …………… Dose: ……… times/day Duration: ……………

☐ both

Supplementary Table S5. Antibiotic susceptibility profile of *K. pneumoniae, E. coli, and St. agalactiae.*

Antibiotic	*K. pneumoniae*	*E. coli*	Antibiotic	*St. agalactiae* MIC (µg/mL)	Interpretation
Amoxicillin	R	R	Benzylpenicillin	≤ 0.12	S
Ticarcillin	R	R	Ampicillin	≤ 0.25	S
Amoxicillin /Clavulanic acid	R	R	Levofloxacin	2	S
Cefazolin	R	R	Moxifloxacin	0.5	S
Cefoxitin	S	R	Clindamycin	≤ 0.25	R
Cefotaxime	R	R	Quinupristin/Dalfopristin	≤ 0.25	S
Imipenem	S	S	Linezolid	2	S
Amikacin	S	S	Vancomycin	≤ 0.5	S
Ciprofloxacin	I	S	Tetracycline	≤ 1	S
Trimethoprim/Sulfamethoxazole	R	S	Tigecycline	≤ 0.12	S
Nitrofurantoin	S	S	Nitrofurantoin	≤ 16	S
Colistin	S	S			
Gentamicin	R	S			
Ceftazidime	R	R			
Nalidixic acid	I	S			
Ciprofloxacin	I	S			
Colistin	S	S			
Aztreonam	R	S			
Nitrofurantoin	S	S			
Fosfomycin	S	S			
Chloramphenicol	S	R			
